# Dissecting the transcriptome landscape of the human fetal neural retina and retinal pigment epithelium by single-cell RNA-seq analysis

**DOI:** 10.1371/journal.pbio.3000365

**Published:** 2019-07-03

**Authors:** Yuqiong Hu, Xiaoye Wang, Boqiang Hu, Yunuo Mao, Yidong Chen, Liying Yan, Jun Yong, Ji Dong, Yuan Wei, Wei Wang, Lu Wen, Jie Qiao, Fuchou Tang

**Affiliations:** 1 Beijing Advanced Innovation Center for Genomics, Department of Obstetrics and Gynecology, Third Hospital, College of Life Sciences, Peking University, Beijing, China; 2 Ministry of Education Key Laboratory of Cell Proliferation and Differentiation, Biomedical Pioneering Innovation Center, Peking University, Beijing, China; 3 Key Laboratory of Assisted Reproduction, Ministry of Education, Beijing, China; 4 Peking-Tsinghua Center for Life Sciences, Academy for Advanced Interdisciplinary Studies, Peking University, Beijing, China; 5 Beijing Key Laboratory of Reproductive Endocrinology and Assisted Reproductive Technology, Beijing, China; University of Michigan, UNITED STATES

## Abstract

The developmental pathway of the neural retina (NR) and retinal pigment epithelium (RPE) has been revealed by extensive research in mice. However, the molecular mechanisms underlying the development of the human NR and RPE, as well as the interactions between these two tissues, have not been well defined. Here, we analyzed 2,421 individual cells from human fetal NR and RPE using single-cell RNA sequencing (RNA-seq) technique and revealed the tightly regulated spatiotemporal gene expression network of human retinal cells. We identified major cell classes of human fetal retina and potential crucial transcription factors for each cell class. We dissected the dynamic expression patterns of visual cycle– and ligand-receptor interaction–related genes in the RPE and NR. Moreover, we provided a map of disease-related genes for human fetal retinal cells and highlighted the importance of retinal progenitor cells as potential targets of inherited retinal diseases. Our findings captured the key in vivo features of the development of the human NR and RPE and offered insightful clues for further functional studies.

## Abstract 2

Single-cell RNA sequencing allows dissection of the transcriptomic landscape of the human fetal neural retina (NR) and retinal pigment epithelium (RPE), revealing a tightly regulated spatiotemporal gene expression network of human retinal cells, as well as dynamic and functional interactions between the NR and RPE.

## Introduction

During the development of the vertebrate embryo, the inside of the optic cup forms the neural retina (NR), whereas the outside becomes the retinal pigment epithelium (RPE) [1–3]. Multipotent retinal progenitor cells (RPCs) are located in the inner layer of the optic cup and give rise to essentially all cell classes in the retina, including six classes of neurons—namely, the output neurons: retinal ganglion cells (RGCs); interneurons such as horizontal cells (HCs), amacrine cells (ACs), and bipolar cells (BCs); and rod and cone photoreceptor cells (PCs), as well as glial cells and Müller glia cells [4–8]. In mice, RGCs are generated at embryonic day (E)8, with a peak at approximately E11. ACs appear at E8, with a peak at E16. BCs are born at E17 [9,10].

Many basic helix-loop-helix (bHLH) transcription factors are expressed in RPCs and are involved in determining retinal cell fate [11–17]. For example, *Pax6*, *Sox2*, and *Vsx2* are transcription factors known to be involved in RPC multipotency [18,19]. *Atoh7* induces the expression of *Pou4f2* and *Isl1*, which are important for the specification and differentiation of RGCs [20,21]. *Foxn4* expression in RPCs is required for AC and HC specification [22]. Foxn4 activates the expression of Neurod1, Neurod4, and Ptf1a to confirm the AC fate [23]. *Rax2* and *Otx2* are involved in the generation of PCs [24]. *Vsx2* and *Ascl1* contribute to the specification of BCs [25]. The overexpression of *Hes1*, *Hes5*, and *Hey2* promotes the Müller glial cell fate [15,26–28]. Although functional studies of the transcription factors mentioned above revealed solid clues for retinal cell fate determination, it is difficult to perform functional experiments on human fetal retina. Thus, the transcription factor profiles of the developing human retina, especially active transcription factors, need to be investigated and could offer helpful clues for the study of human retina development in retinal organoids [29–32].

In humans, the analysis of a histological collection of human eyes identified the sequence of retinal layer development [33]. Immunohistochemical staining for transcription factor expression in human embryos at 6–12 weeks (W) showed the division of the early NRs into the KI67 (a marker of proliferating cells)-positive outer layer and the Tuj1 (a neuronal marker)-positive inner layer. Additionally, the expression patterns of known eye-field transcription factors and related transcription factors in these two layers have also been revealed [34]. Although the histological analysis of the developing human retina has helped researchers understand the structures and morphology of retinal cells, detailed molecular evidence for the development of human retina is lacking. Numerous studies of the human NR and RPE transcriptome revealed the gene expression in these two tissues [35–37]. Hoshino and colleagues and Aldiri and colleagues analyzed bulk RNA sequencing (RNA-seq) data of human fetal retina [38,39]. More specific expression profiles of each human fetal retinal cell class need to be investigated.

Single-cell RNA-seq analysis could satisfy the need of higher specificity. Macosko and colleagues and Shekar and colleagues performed single-cell RNA-seq on mouse retina after birth using Drop-seq [40,41]. They offered rich resources for mouse retina studies. There are several studies on the human retina preprinted at bioRxiv [42–44]. Liang, Qingnan, and colleagues and Lukowski, Samuel, and colleagues completed the adult retina single-cell transcriptome atlas, and Lu, Yufeng, and colleagues provided a resource for the fetal human retina at the stages after 12 W. However, none of them discussed the development of fetal retinal cells at early stages (before 12 W) or the interaction of NR and RPE. Our work is the first to dissect the transcriptome landscape of NR and RPE before 12 W.

We performed single-cell RNA-seq using a previously described modified single-cell tagged reverse transcription (STRT) method [45–48] on individual cells collected from the human fetal NR and RPE at 5–24 W, following receipt of ethical approval, to address these questions. We identified the major cell classes in the NR and RPE, providing the single-cell transcriptome landscape of each cell class. Importantly, we dissected the temporal order of the generation of human retinal cells. Through a systematic bioinformatics analysis, we revealed important transcription factors with high activity in regulatory networks required for the development of the NR and RPE. Moreover, we revealed dynamic changes in the transcriptome features of RGCs and RPE cells. We explored the developmental period at which the visual cycle might occur based on the single-cell transcriptome features of PCs and RPE cells, as well as other interactions of these two cell classes. Furthermore, we mapped disease-related genes in our data set to explore potential targets of inherited retinal diseases. Thus, we offer a rich resource for studying the human NR and PRE. The expression patterns of single-cell RNA-seq data in the present study are available at http://49.4.93.68:30004/.

## Results

### Single-cell RNA-seq profiling of the human fetal NR and RPE

**Fig 1:**
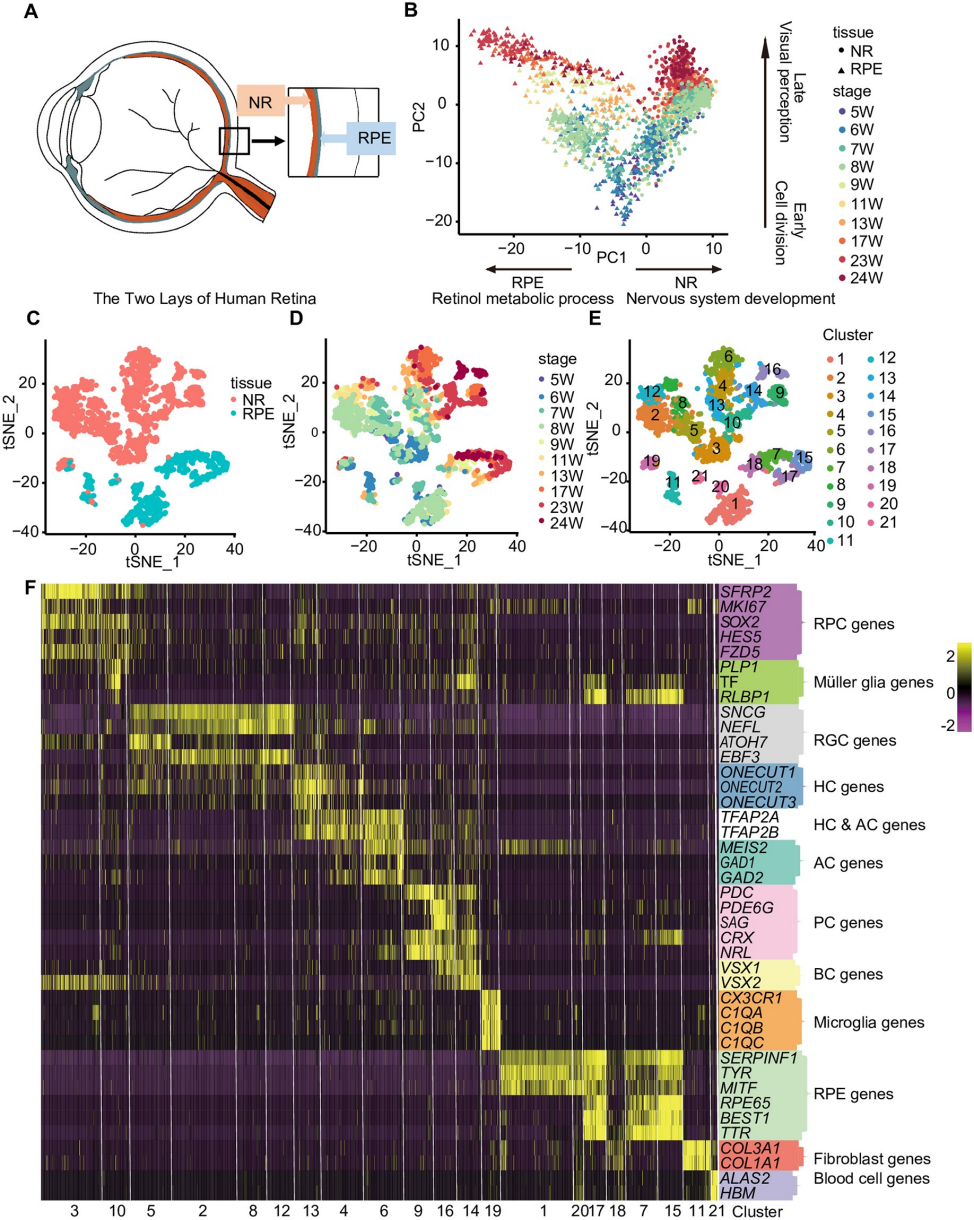
Single-cell RNA-seq transcriptome profiling of human fetal NR and RPE. (A) The schematic of NR lay and RPE lay in human eyes. (B) PCA of single cells collected from the NR and RPE. Tissue types are shown as different shapes, and stages are shown in different colors. (C) t-SNE plot showing the tissue types of the cells. Tissue types are shown in different colors. (D) t-SNE plot showing the stages of the cells. Stages are shown in different colors. (E) Clustering of 2,421 single-cell expression profiles into 21 clusters. Clusters are shown in different colors. (F) Heatmap showing the expression patterns of known retinal cell and non–retinal cell markers in individual cells of each cluster. AC, amacrine cell; BC, bipolar cell; HC, horizontal cell; NR, neural retina; PC, photoreceptor cell; PCA, principal component analysis; RGC, retinal ganglion cell; RPC, retinal progenitor cell; RPE, retinal pigment epithelium; t-SNE, t-distributed stochastic neighbor embedding; W, week.

We performed single-cell RNA-seq analysis of individual cells isolated from the human fetal NR and RPE ranging from 5 to 24 W (10 developmental stages) to obtain transcriptional maps of these two layers (Fig 1A). After quality control (gene number ≥ 1,000, unique molecular identifier [UMI] ≥ 10,000), 2,421 cells were retained, and an average of 4,631 expressed genes (transcripts per million [TPM] > 0) (S1 Fig) and 129,105 transcripts were detected in each individual cell (S1 Fig). We did not detect significant batch effects in these experiments (S1 Fig).

We performed an unbiased principal component analysis (PCA) of these 2,421 cells using highly variable genes to examine the global transcriptome patterns in the single-cell RNA-seq data (Fig 1B). The first segregating factor was different tissues, as cells were divided into two groups consistent with the tissue layers from which they were collected. The positive genes in PC1 were enriched for gene ontology (GO) terms such as nervous system development (*P* value = 6.2E-09), and the negative genes in PC1 were enriched for GO terms such as retinol metabolic process (*P* value = 1.9E-04). The second segregating factor was the developmental stage. Cells were ordered in a time course from the early to the late stage. The positive genes in PC2 were related to visual perception (*P* value = 2.3E-12), and the negative genes were related to cell division (*P* value = 2.4E-10) (S1 Table).

We reduced the 18 statistically significant principal components in the PCA to two dimensions using t-distributed stochastic neighbor embedding (t-SNE) [40] to define the cell identities. These 2,421 cells were divided into 21 distinct clusters (Fig 1E). Cells from different layers were clearly segregated and ordered in a time-dependent manner (Fig 1C and 1D). Based on the specific expression of known cell type–specific markers [49] for the NR and RPE (Fig 1F), we identified major cell classes of human fetal retina—namely, RPE cells expressing *SERPINF1*, *TYR*, *MITF*, *RPE65*, *BEST1*, and *TTR*; RPCs expressing *SFRP2*, *MKI67*, *SOX2*, *HES5*, and *FZD5*; RGCs expressing gamma synuclein (*SNCG*), *NEFL*, *ATOH7*, and *EBF3*; HCs expressing *ONECUT1/2/3*; ACs expressing *MEIS2*, *GAD1*, and *GAD2*; BCs expressing *VSX1* and *VSX2*; PCs expressing *PDC*, *PDE6G*, *SAG*, *CRX*, and *NRL*; Müller glia expressing *PLP1*, *TF*, and *RLBP1*; microglia expressing *CX3CR1*, *C1QA*, *C1QB*, and *C1QC*; fibroblasts expressing *COL3A1* and *COL1A1*; and a few blood cells expressing *ALAS2* and *HBM*. Markers of each cluster and their GO analysis results were provided in S2 and S3 Tables.

**Fig 2:**
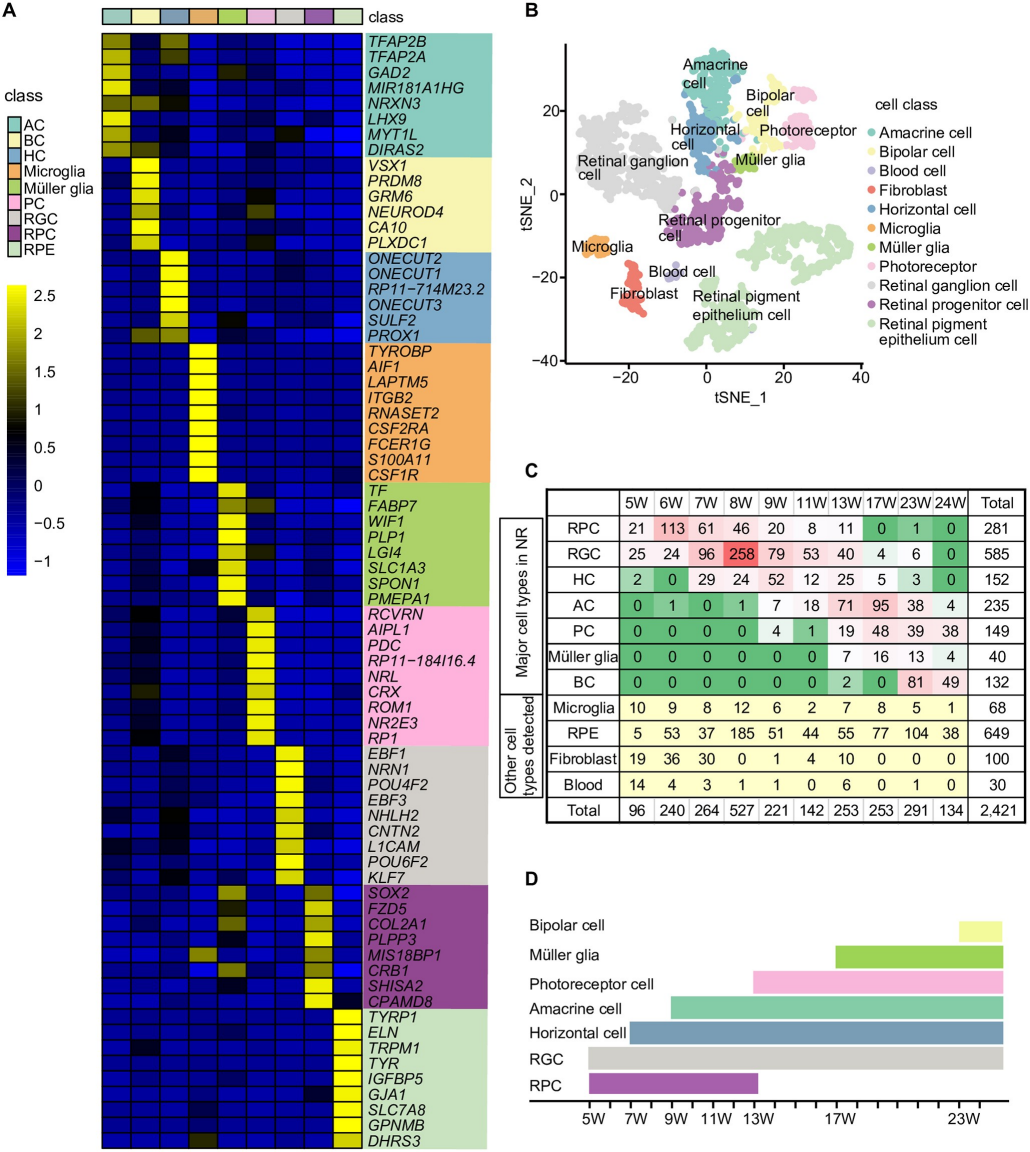
Cell classes in the NR and the temporal order of the generation of human retinal cells. (A) Heatmap showing the mean expression levels of representative and enriched genes for each class of retinal cell in the NR. The color key from blue to yellow indicates low to high gene expression levels, respectively. Expression levels are quantified as log2 (mean TPM / 100 + 1). (B) t-SNE map of defined cell classes in the NR and RPE. Cell classes are shown in different colors. (C) Numbers of each cell class detected. (D) The temporal order of the generation of human retinal cells. AC, amacrine cell; BC, bipolar cell; HC, horizontal cell; NR, neural retina; PC, photoreceptor cell; RGC, retinal ganglion cell; RPC, retinal progenitor cell; RPE, retinal pigment epithelium; TPM, transcripts per million; t-SNE, t-distributed stochastic neighbor embedding; W, week.

We further analyzed clusters containing various cell classes to obtain a more accurate identity for each single cell (S4 Table). For example, cluster 16 contained both *VSX1* [50] (BC marker)-positive and *NRL* [51] (a rod transcription factor)-positive cells (Fig 1F). We performed t-SNE analysis on the cells in cluster 16 to define BCs and PCs in cluster 16 (S2 Fig). Cells in cluster 16 could be divided into two subclasses, one expressing BC-enriched genes *CHN2* and *SCN3A* and the other expressing PC markers *PDC* and *AIPL1*. The same strategies were performed for cluster 10 (containing RPCs and Müller glia) (S2 Fig), cluster 4 (containing HCs and ACs), and cluster 3 (containing RPC subclasses) (S2 Fig). Subsequently, each single cell was assigned a potential identity for further analyses (Fig 2B, S5 Table). Cell class–specific markers were identified using the FindMarker function in Seurat (Fig 2A and S4 Table). According to the cell class–specific markers, we inferred cells in the PC group as rod photoreceptors because they expressed earlier markers of rod photoreceptors, such as *NRL* and *ROM1* [52]. Besides, we found that *NTRK1*, which was reported to be expressed in injured RGCs [53,54], was one of the markers in human fetal HCs. We detected the expression of *NTRK1* in *ONECUT2* (a marker of HCs)-positive cells by RNAscope assay (S3 Fig).

We detected four subclasses of RPCs (S2 Fig). The differentially expressed gene (DEG) analysis showed that one RPC subclass had relatively higher expression levels of RPC-enriched genes *SFRP2*, *SHISA2*, and *CPAMD8*. One subclass was highly proliferative, expressing *TOP2A* and *MKI67*. RGC markers, such as *NEFM*, *PRPH*, *NEFL*, *ISL1*, and *GAP43*, were expressed in one RPC subclass. We detected a small group of RPCs expressing *C1QA*, *CD74*, and *HLA-DRA*.

### The developmental order of the generations of the human NR cells

The single-cell RNA-seq analysis was used to identify the different classes of cells in the developing NR and markers for each retinal cell class. A GO analysis of markers for each cell cluster indicated the potential states of each cell class (S2 and S3 Tables). For example, RPCs were grouped in cluster 3, and the GO analysis showed that these cells were enriched for cell division processes (*P* value = 1.8E-20). Clusters 2, 5, 8, and 12 were RGCs that primarily participated in nervous system and axon development. Clusters 13 and 4 were HCs. Markers of cluster 13 were related to neuron projection development (*P* value = 4.1E-05) and retinal layer formation (*P* value = 5.1E-05). Cluster 6 contained ACs that participate in neurotransmitter secretion (*P* value = 5.0E-12) and chemical synaptic transmission (*P* value = 4.3E-09). Markers of cluster 9 were significantly related to PC maintenance (*P* value = 4.9E-07). BCs in cluster 14 were involved in visual perception (*P* value = 2.8E-09), response to stimulus (*P* value = 1.1E-05), and retinal homeostasis (*P* value = 3.8E-05).

We summarized the number of cells of each class at each stage based on the data in S5 Table to determine the time at which each cell class in the NR was generated (Fig 2C and 2D). RPCs and RGCs were already observed at 5 W. The proportion of RPCs peaked at 6 W, and the highest proportion of RGCs was observed at 8 W. Subsequently, HCs developed (reached a peak at 9 W), followed by ACs (reached a peak at 17 W), PCs, Müller glia cells, and BCs.

**Fig 3:**
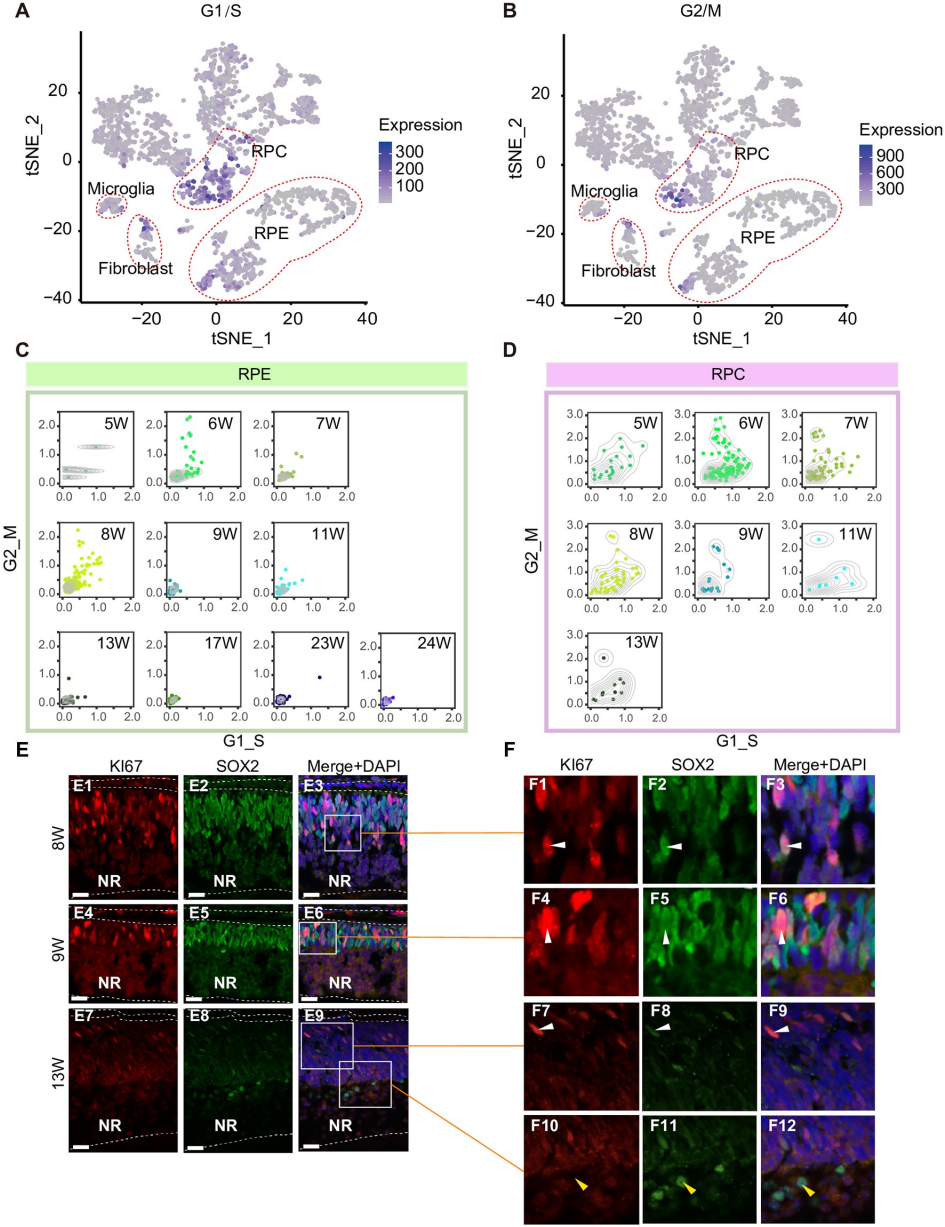
Cell cycle states of cells in the developing human NR and RPE. (A) Relative expression of gene sets associated with G1/S phases for each cell mapped on the t-SNE plot. The color key from gray to blue indicates low to high gene expression levels, respectively. Expression levels are quantified as the mean TPM of each gene set. (B) Relative expression of gene sets associated with G2/M phases for each cell mapped on the t-SNE plot. The color key from gray to blue indicates low to high gene expression levels, respectively. Expression levels are quantified as the mean TPM of each gene set. (C) Density map showing the cell cycle state of RPE cells at each developmental period. (D) Density map showing the cell cycle state of RPCs at each developmental period. (E) Validation of proliferating cells in the human retina by immunofluorescence staining for the proliferation marker KI67 and SOX2, which labels RPCs and Müller glia cells. Scale bars are 25 μm. (F) Higher-magnification images of selected regions shown in Fig 3E. NR, neural retina; RPC, retinal progenitor cell; RPE, retinal pigment epithelium; TPM, transcripts per million; t-SNE, t-distributed stochastic neighbor embedding; W, week.

Next, we analyzed the cell cycle features of each cell class we identified (S6 Table) [55]. At 5 and 6 W, fibroblasts, microglia, and RPE cells were actively proliferating (Fig 3A–3C, S4 Fig). RPCs in the NR were actively proliferating (Fig 3A, 3B and 3D, S4 Fig). We used KI67, the marker of proliferating cells, and the RPC-enriched gene SOX2 to evaluate the location and state of RPCs in the developing human retina (Fig 3E and 3F). KI67^+^ and SOX2^+^ cells were located in the outer layer of the retina. The density of KI67^+^ cells in the NR decreased as retinal development proceeded. We detected two classes of SOX2^+^ cells in the NR at 13 W. One SOX2^+^ cell class had a spindle-shaped nucleus resembling RPCs at 8 W and 9 W; this class of cell was located at the outer nuclear layer (ONL). The other SOX2^+^ cell class had a round-shaped nucleus and was located in the inner nuclear layer (INL). We postulated that the round SOX2^+^ cells in the INL were Müller glia cells.

### The expression patterns of transcription factors in the human NR and RPE

Transcription factors play important roles in eye development. To identify transcription factors with high activity, we applied a method called single-cell regulatory network inference and clustering (SCENIC) [56], which evaluates the activity of the gene regulatory networks (GRNs) in each cell. The t-SNE analysis of the binary regulon activity matrix (S5 Fig) was consistent with the t-SNE analysis based on expression matrices in Fig 2B.

**Fig 4:**
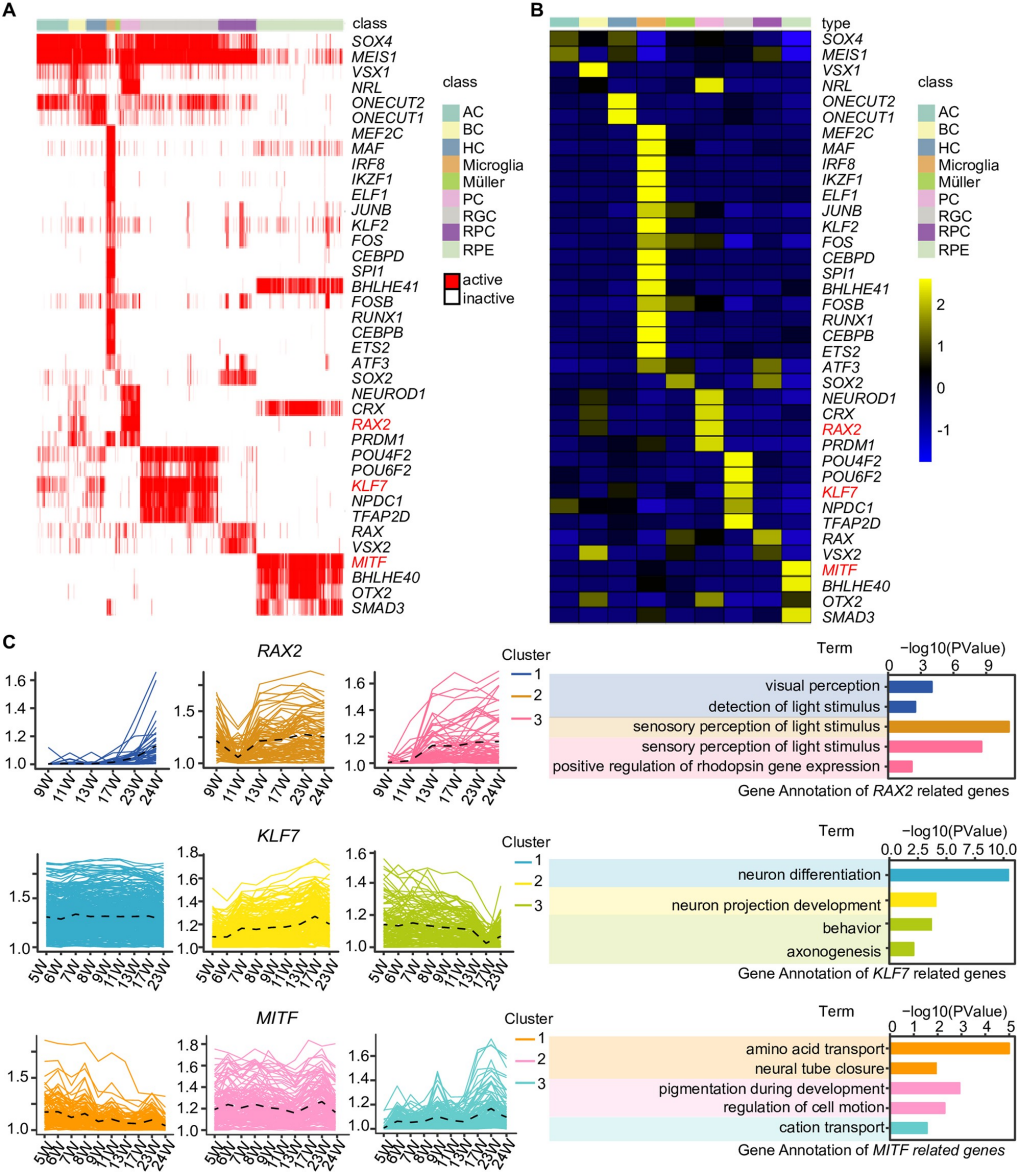
The expression of important transcription factors in the human fetal NR and RPE. (A) SCENIC results for the human fetal NR and RPE. Heatmap showing the active and expressed transcription factors in each cell class. The states of the transcription factors in each cell class are indicated in red (active) and white (inactive). (B) Heatmap showing the mean expression of the active transcription factors in each cell class. Expression levels are indicated with colors. (C) The expression patterns of representative transcription factors’ target genes across developmental stages (left) and the GO analysis of these genes (right). AC, amacrine cell; BC, bipolar cell; GO, gene ontology; HC, horizontal cell; NR, neural retina; PC, photoreceptor cell; RGC, retinal ganglion cell; RPC, retinal progenitor cell; RPE, retinal pigment epithelium; SCENIC, single-cell regulatory network inference and clustering; W, week.

In the present study, transcription factors that were active among more than 50% of cells in a particular cell class were retained. The transcription factors with high activities were selected by the AUCell algorithm (Fig 4A and 4B), and they were defined as active transcription factors. In the NR, RPCs expressed differentiation-related genes, including *SOX2*, *RAX*, and *VSX2*. *POU4F2*, *POU6F2*, *KLF7*, *NPDC1*, and *TFAP2D* were active in RGCs. *SOX4* and *MEIS1* were active in ACs. *ONECUT1* and *ONECUT2* were active in developing HCs. *NEUROD1*, *NRL*, *CRX*, *RAX2*, and *PRDM1* were primarily expressed in PCs. *VSX1* and *VSX2* were expressed at high levels in BCs. In the RPE, *MITF*, *BHLHE40*, *OTX2*, and *SMAD3* were active.

Using SCENIC, the target genes of each transcription factor could be identified (Fig 4C). *RAX2* was active in PCs. During the development of the retina, the increased target genes of *RAX2* were involved in visual perception and detection of light stimulus, which is consistent with the function of PCs. *KLF7* was active in RGCs. A set of *KLF7* target genes was constantly expressed and related to neuron projection development. *MITF* was uniquely active and expressed in RPE, which targeted amino acid transport and neural tube closure–related genes at early stages and cation transport genes at late stages. *MITF* also targeted genes that were related to pigmentation during development and regulation of cell motion.

*SOX2* was mainly expressed in RPCs and Müller glia cells. *POU4F2* was an RGC-specific transcription factor. *NEUROD1* was detected in various retinal cells but was mainly expressed in PCs (S5 Fig). We verified these representative transcription factors by immunofluorescence staining and determined their distribution and expression in structures of the human fetal NR (S5 Fig). At 9 and 13 W, RPCs were SOX2^+^ and NEUROD1^−^. RPCs were located in the outer layers of the NR (near the RPE) at 9 W and then were shifted away from near the RPE toward INL at 13 W. Few RPCs were detected at 23 W, whereas Müller glia cells were observed in the INL of the NR at 13 and 23 W. At 13 W, the NEUROD1^+^SOX2^−^ cells adjacent to the RPE were likely PCs. We detected SOX2^+^NEUROD1^+^ cells at 23 W, and they might be newborn neurons. The NEUROD1^+^SOX2^−^ cells located next to Müller glia cells might be BCs. A majority of the BRN3B^+^ (gene *POU4F2*) (S5 Fig) cells were located farthest away from the RPE, and these cells were probably RGCs. At 9 W, the BRN3B^−^NEUROD1^+^ TFAP2B^+^ cells distributed among the RGCs might be ACs.

### The dynamic changes in the transcriptome of human fetal RGCs

**Fig 5:**
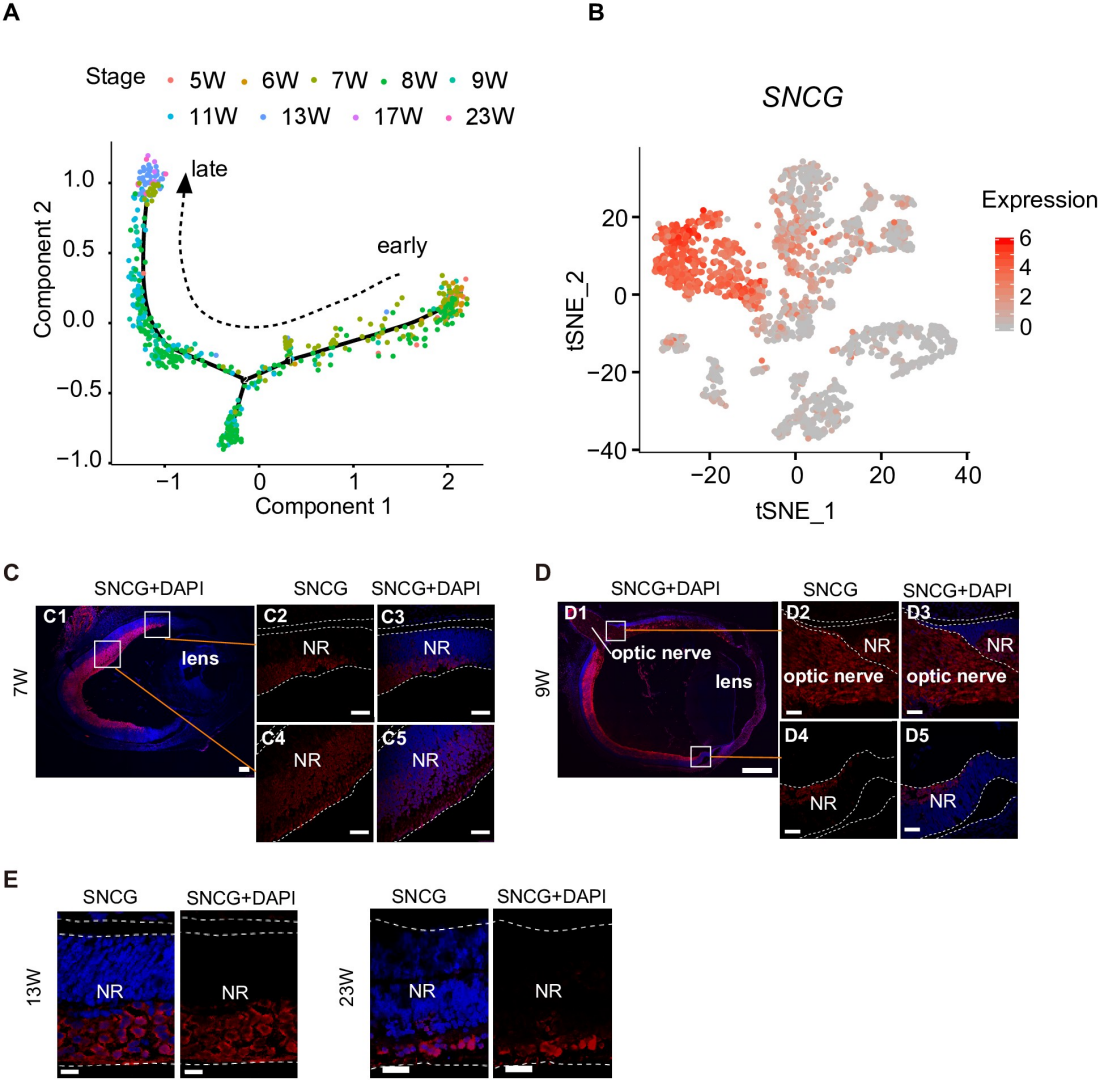
The development of RGCs and their dynamic transcriptome features. (A) Developmental pseudotime of RGCs calculated using Monocle2. Sampling stages are shown in different colors. (B) Expression of the RGC-enriched gene *SNCG* mapped on the t-SNE plot. (C, D, and E) Stages and locations of RGCs in the developing retina. RGCs are labeled by SNCG. Higher-magnification images of selected regions are shown in (C2-C5) and (D2-D7). White bars represent 100 μm in (C1), 500 μm in (D1), 25 μm in (E), and 50 μm in (C2-C5) and (D2-D5). NR, neural retina; RGC, retinal ganglion cell; SNCG, gamma synuclein; t-SNE, t-distributed stochastic neighbor embedding; W, week.

We obtained 585 RGCs from 5 to 23 W. We performed pseudotemporal ordering of the single cells using Monocle2 [57] to reveal the dynamic changes in the transcriptome of the developing human RGCs. We applied the extreme gradient boosting (xgboost) R package v0.7.0 training a model to identify any different RGCs based on the expression levels of all genes. The genes with the top 10 highest importance scores in this model were selected to obtain a pseudo–developmental time using the discriminative dimensionality reduction tree (DDRTree) algorithm of the Monocle package combined with the trajectory information (S7 Table). Based on our data, the pseudotemporal order of the cells matched their actual developmental stages (Fig 5A). Using the genSmoothCurves function, we analyzed the dynamic expression levels of RGC-enriched genes.

During the RGC development, the expression levels of the bHLH genes *ATOH7* and *NEUROD1*, the cell adhesion molecule *CNTN2*, and progenitor markers *SFRP2* and *ID1* decreased. We detected increased expression levels of RGC master genes, such as *FXYD7*, *MEG3*, *NEFL*, *NEFM*, *SNCA*, *MAPT*, *NTM*, and *RXRG*, in developing RGCs. The expression levels of *ELAVL4*, *GAP43*, *PFN1*, *PRPH*, *RTN1*, and *TUBB2A* remained constant in RGCs at different stages (S6 Fig).

According to the results of the GO analysis, genes specific to early RGCs were involved in the response to BMP (*P* value = 2.0E-07). Genes specific to late RGCs participated in the energy derivation by oxidation of organic compounds (*P* value = 4.9E-11) and fatty acid beta-oxidation (*P* value = 3.1E-05) (S6 Fig).

We stained fixed human fetal retinas for the RGC marker SNCG to obtain spatial information about RGC generation in the human retina. Immunostaining revealed the gradual generation of RGCs from the optic nerve head toward the periphery, and RGCs were essentially spread throughout the ganglion cell layer (GCL) at approximately 9 W (Fig 5D). The proportion and density of RGCs gradually decreased during the development of the human retina (Fig 5E). We also detected SNCG proteins in axons located in the optic nerve (Fig 5D1–5D3).

### The development of human RPE cells

**Fig 6:**
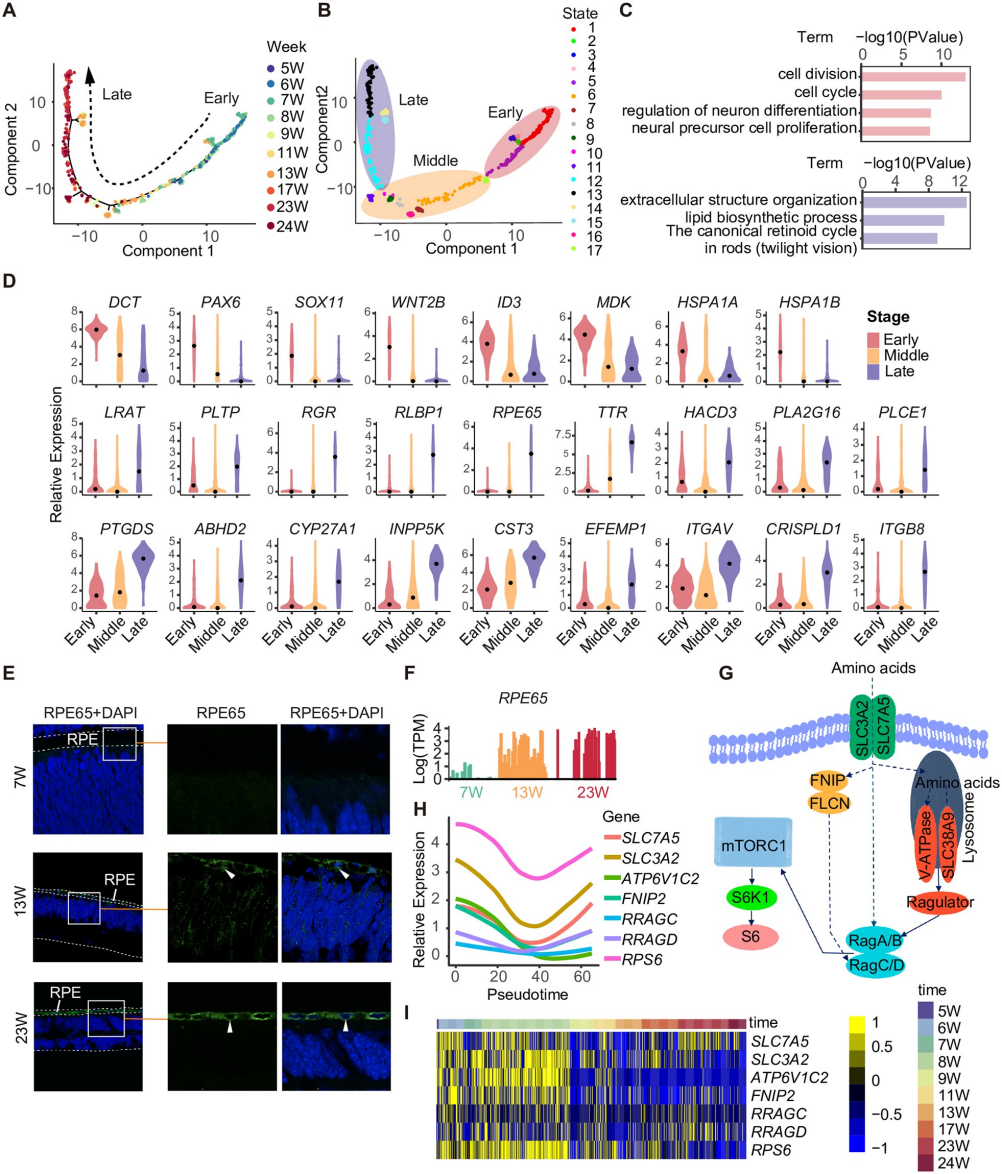
The development of the RPE. (A) Developmental pseudotime of RPE cells calculated using Monocle2. Sampling stages are shown in different colors. (B) The state classification of RPE cells. (C) The GO analysis of the down-regulated genes and up-regulated genes during the development of RPE cells. (D) The expression patterns of representative genes of the early and late RPE cells. (E) Validation of RPE65 expression in the developing RPE at the protein level. White bars represent 25 μm. Representative cells are indicated with white arrows. (F) Bar plot showing the expression of *RPE65* in RPE cells at 7, 13, and 23 W. Stages are shown in different colors. (G) The schematic of amino acids signaling pathway. (H) The expression tendency of amino acids signaling pathway–related genes in RPE cells. (I) The heatmap showing expression patterns of amino acids signaling pathway–related genes in RPE cells. GO, gene ontology; RPE, retinal pigment epithelium; TPM, transcripts per million; W, week.

We dissected the dynamic changes in the transcriptome of RPE cells using Monocle2. RPE cells were ordered in pseudotime consistent with the actual developmental stages (Fig 6A). The states of RPE cells could be divided into early, middle, and late states. We applied the R package (cummeRbund) to group RPE high-variable genes (S7 Fig, S8 Table). Nine clusters of RPE high-variable genes were identified. The down-regulated genes were related to cell division (Midkine [*MDK*], *HSPA1A*, and *HSPA1B*), cell cycle, regulation of neuron differentiation (*ID3*), and neural precursor cell proliferation (*DCT*, *PAX6*, *SOX11*, and *WNT2B*). The up-regulated genes were related to extracellular structure organization (*CST3*, *EFEMP1*, *ITGAV*, *CRISPLD1*, and *ITGB8*), lipid biosynthetic process (*HACD3*, *PLA2G16*, *PLCE1*, *PTGDS*, *ABHD2*, *CYP27A1*, and *INPP5K*), and the canonical retinoid cycle in rods (*LRAT*, *PLTP*, *RGR*, *PLBP1*, *RPE65*, and *TTR*) (Fig 6C and 6D). The expression of RPE65 was validated by immunofluorescence staining (Fig 6E and 6F). In addition, Kyoto Encyclopedia of Genes and Genomes (KEGG) pathway mapping of RPE-enriched genes showed that amino acid signaling pathway–related genes were highly expressed at early stages (Fig 6G–6I).

### Clues about interactions of RPE cells with photoreceptors

**Fig 7:**
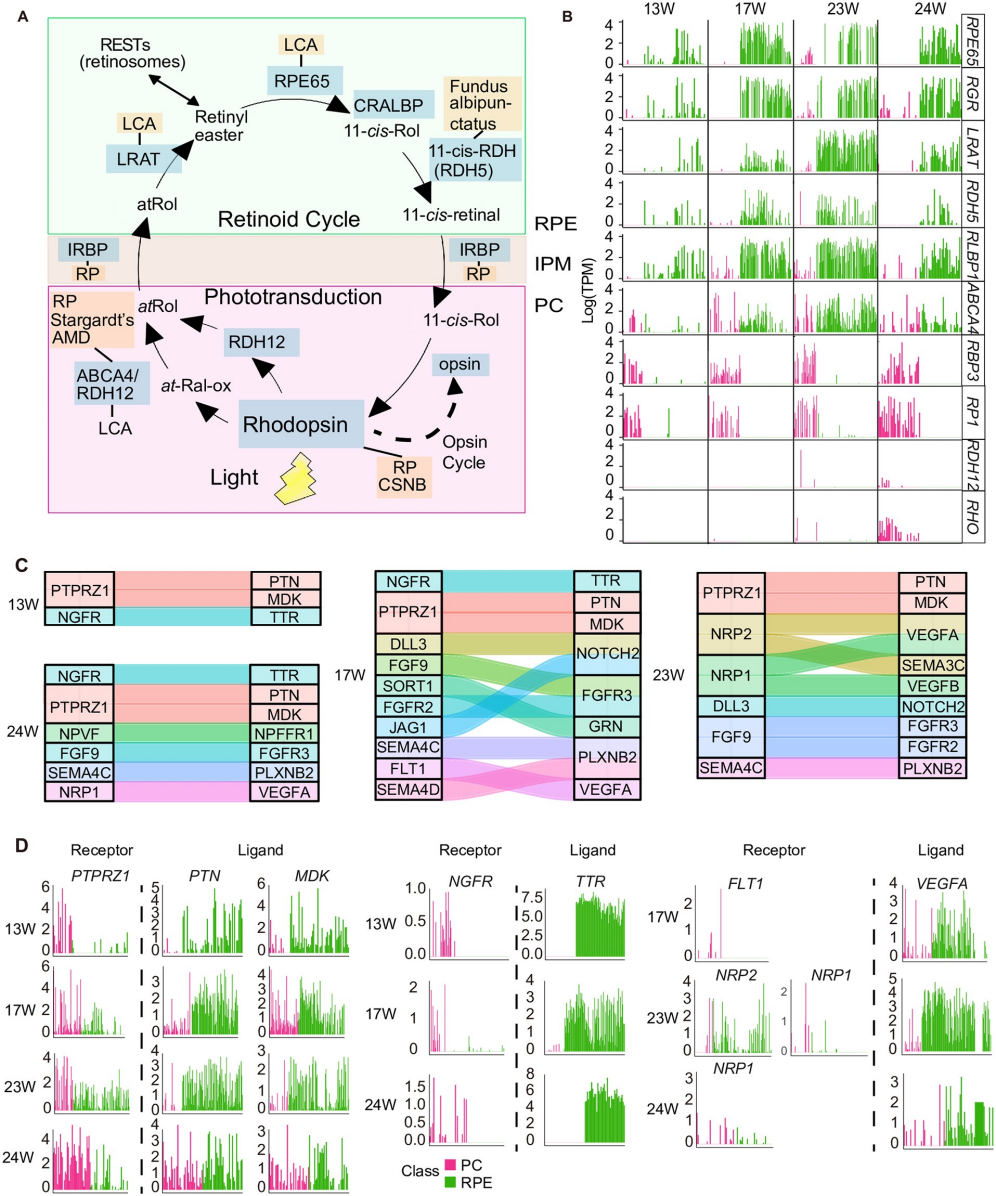
Clues about the interaction of RPE cells with PCs. (A) The visual (retinoid) cycle and phototransduction in RPE cells and PCs. (B) The expression of visual cycle–related genes in PCs and RPE cells. Cell classes are shown in different colors. (C) The receptor–ligand pairs identified by CellPhoneDB. (D) Barplot showing the expression levels of representative receptor–ligand pairs. AMD, age-related macular degeneration; CRALBP, cellular retinaldehyde binding protein; IPM, interphotoreceptor matrix; PC, photoreceptor cell; RPE, retinal pigment epithelium; TPM, transcripts per million; W, week.

To identify the interactions between RPE cells and PCs in fetal human retina, we explored the expression levels of visual cycle–and phototransduction-related genes [58]. First, we analyzed the states of PCs using PCA, and these cells were divided into three subgroups (S7 Fig). Groups 2 and 3, comprising cells from retinas at 23 and 24 W, were more mature PCs, as they expressed not only PC-enriched genes, such as *NRL*, *AIPL*, *RP1*, *GNGT2*, *NEUROD1*, *PDC*, *RXRG*, *CRX*, and *PDE6G*, but also genes related to phototransduction, such as *RHO*, *SAG*, *GNGT1*, and *CNGA1* (S7 Fig). Group 1 seemed to be less mature PCs because of their lower expression levels of visual perception genes, and most of them were collected from earlier stages. *SAG*, *GNGT1*, and *CNGA1* are involved in the rhodopsin-mediated signaling pathway. Moreover, we detected *ABCA4*, *RBP3*, and *RHO* in PCs (Fig 7B). At 24 W, more PCs expressed *RHO*, a rhodopsin expressed in rod cells that is also involved in the rhodopsin-signaling pathway. Light transduction was reported to begin with photon absorption by rhodopsin [59]. Based on our results, light transduction might occur at 24 W. In RPE cells, *RPE65*, *RDH5*, *LRAT*, *RGR*, and *RLBP1* were expressed at 13 W. In the visual cycle, RGR (also known as RPE-retinal G-protein-coupled receptor) is used to isomerize all-*trans*-retinal to 11-*cis*-retinal, and then the 11-*cis*-retinal is delivered back to PCs. RPE65, RDH5, and LRAT enzymes catalyze the reaction from all-*trans*-retinol to 11-*cis*-retinal. RLBP1 (also known as the cellular retinaldehyde binding protein [CRALBP]) speeds up the reaction [60] (Fig 7A). Taken together, the visual cycle was probably initiated as early as 13 W, long before visual perception began.

In addition, using CellPhoneDB [61] (www.CellPhoneDB.org), we found more clues about interactions between RPE cells and PCs (Fig 7C and 7D). For example, we detected two neurotrophic factors *MDK* and *PTN* (pleiotrophin) in RPE cells. PTN and MDK are involved in inducing and stimulating neuronal differentiation. Receptor protein tyrosine phosphatase type Z (*PTPRZ1*), a shared receptor of *MDK* and *PTN*, was highly expressed in PCs. The binding of MDK to PTPRZ1 is important for MDK-dependent survival of embryonic neurons. The interaction of PTN with PTPRZ1 is known to play an important role in cell–cell adhesion, cell motility and migration, cell division, and epithelial–mesenchymal transition [62]. The interactions of PTPRZ1 with both MDK and PTN might be key for the development of PCs and RPE cells.

### Profiling of inherited retinal disease–related genes in human fetal retinal cells

**Fig 8:**
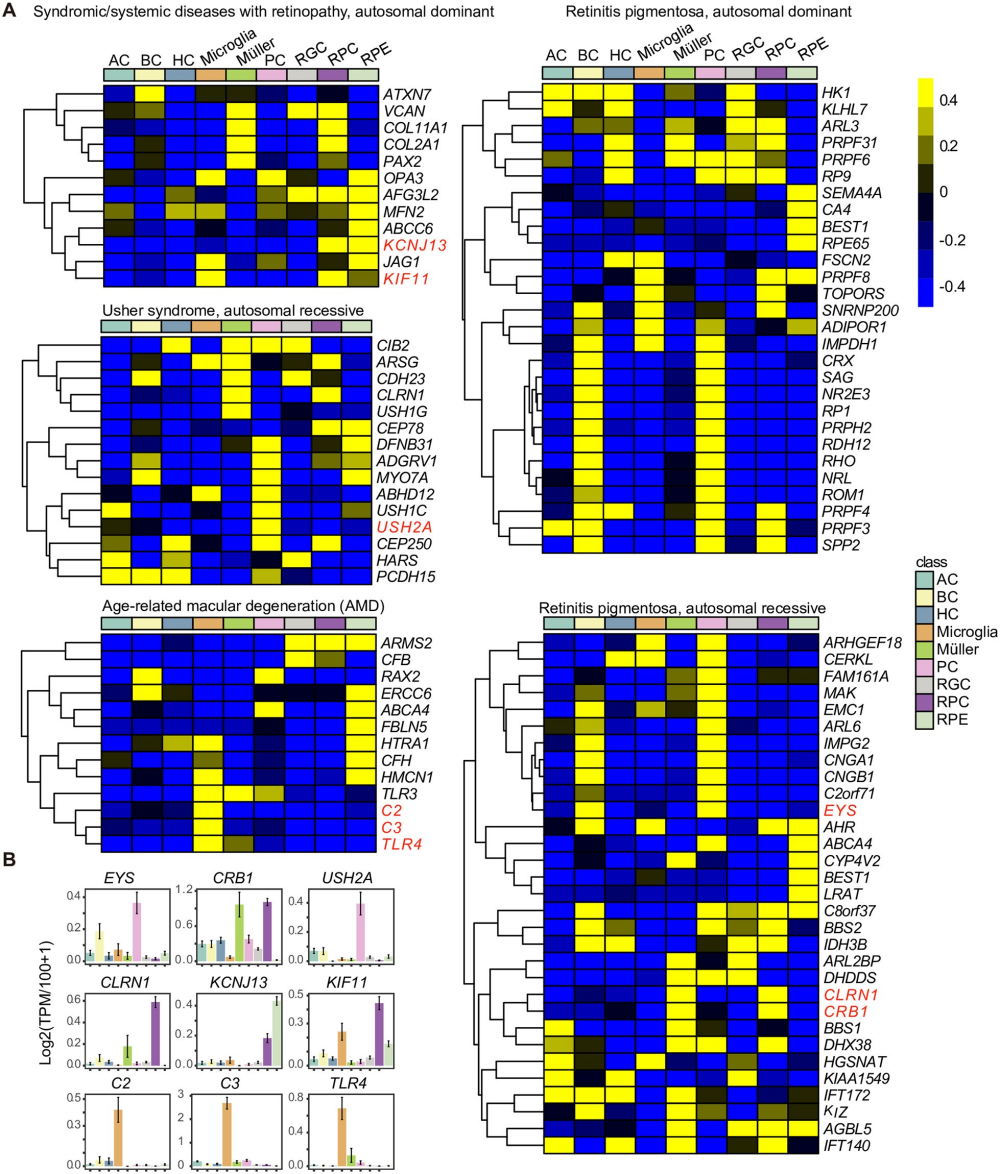
Inherited retinal disease–related genes in human fetal retinal cells. (A) Cluster heatmap of inherited retinal disease–related genes in human fetal retinal cells. (B) Bar plots of representative genes. Cell classes are shown in different colors. AC, amacrine cell; BC, bipolar cell; HC, horizontal cell; PC, photoreceptor cell; RGC, retinal ganglion cell; RPC, retinal progenitor cell; RPE, retinal pigment epithelium; TPM, transcripts per million.

RetNet (https://sph.uth.edu/retnet/) has provided genes whose mutations cause inherited retinal diseases. We wondered whether human fetal retinal cells could be disease targets. In the human fetal retina, most inherited retinal disease–related genes were enriched in PCs, BCs, and RPE cells, such as autosomal dominant/recessive retinitis pigmentosa, X-linked macular degeneration, and other autosomal dominant/recessive retinopathy (Fig 8A and S8 Fig). A previous study showed that *USH2A*, *EYS*, and *CRB1* were the top three genes contributing to inherited retinal dystrophy in Chinese patients [63]. In our data set, *USH2A* was mainly enriched in PCs. *EYS* was highly expressed in BCs and PCs. Interestingly, *CRB1* was enriched not only in Müller glia cells but also in RPCs. In addition, RPCs were also targets of *CLRN1*, *KCNJ13*, and *KIF11* (Fig 8B). As RPCs are important for retinogenesis, mutations in disease-related genes in RPCs could be key for the study of disease processes.

Microglia played a role in the pathogenesis of human age-related macular degeneration (AMD) [64,65]. In mice, microglia were reported to express genes associated with AMD [49]. In addition to *TLR4*, which was also expressed in mouse microglia, we found that C2 and C3 were enriched in human microglia (Fig 8A and 8B).

## Discussion

To our knowledge, the present study is the first to highlight the transcriptome landscape of the human fetal NR and RPE in parallel at a single-cell resolution. In general, the gene expression networks of the NR and the RPE showed substantial differences. NR-specific genes were related to nervous system development, whereas RPE-specific genes were related to retinol metabolism. However, both tissues underwent processes ranging from active cell proliferation to the termination of proliferation and the stepwise maturation of visual perception throughout development; these results indicated their functional interactions at late stages. The NR and RPE have been reported to codifferentiate together. Once the connection is lost, the single-layered RPE forms a multilayered retina-like structure [66].

In the present study, all 2,421 cells were clustered into well-defined cell classes based on known markers identified through mouse models and adult human retina studies, indicating the conserved feature of these markers among mice and humans. Then, we revealed markers of human retinal cells and dissected the temporal order of the generation of retinal cells in vivo. In humans, RGCs were already present at 5 W and peaked at 8 W. HCs were detected at 7 W and peaked at 9 W. Subsequently, ACs peaked at 17 W, followed by PCs. BCs and Müller glia cells were observed last.

We compared the markers of fetal NR cells with the markers of adult NR cells (S9 Fig) [42]. We found that most top 15 markers (10 out of 15) in adult human BCs were also detected in fetal human BCs, such as *TRPM1*, *LRTM1*, *TMEM215*, *PLXDC1*, *NETO1*, *CA10*, *ST18*, *CHN2*, *SLC4A10*, and *KCNMA1*. The top 30 RGC markers in adult retina showed less specificity in fetal RGCs. However, some other RGC markers were shared in adult and fetal retina, such as *GAP43*, *PRPH*, *SYT4*, *INA*, and *NEFL*. This finding might due to the fact that BCs detected in our study were from late embryos, whereas RGCs were from early embryos. Cell states in late embryos might be more similar to mature cells than those in early embryos. Our findings highlighted the differences between retinal cells at early stages and those at late embryonic stages or in adult retina. In addition, fetal PCs in our study highly expressed adult rod genes. The adult cone genes were seldom detected.

We compared our single-cell RNA-seq data to the bulk transcriptomic studies of Hoshino and colleagues and revealed more details on transcriptome dynamics of fetal human retina (S9 Fig). In general, the DEGs between retinas at early and late time points found by bulk RNA-seq analysis could mapped on specific cell classes in our single-cell data. However, we found that some DEGs up-regulated at later time points might be expressed in the same cell class at early time points. For example, *PROX1*, an important transcription factor for generating HC, was reported to begin expressing after fetal day (D) 67 (9.5 W), whereas in our study, *PROX1* could be detected in HCs as early as 8 W. Although our study could dissect more transcriptome features at single-cell resolution, it has limitations. As mentioned above, fetal PCs in our study highly expressed rod genes, and seldom were cone genes detected. In the bulk RNA-seq data, the cone gene *GNAT2* was up-regulated starting at D80 (11.4 W). Cone genes might be detected if we expanded the cell numbers. We evaluated the specificity of each human fetal retinal cell class–enriched gene in retinas of macaque [67] and mouse [40] (S10 Fig). We found that more human fetal retinal cell class–enriched genes demonstrated enrichment in mouse retina than did those in macaque retina.

One important issue in retinal development is the determination of retinal cell fate by transcription factors. We provided a key profile of transcription factors that were active and cell class–specific in developing human retinal and RPE cells. The functions of transcription factors in the developing human retina are difficult to verify; thus, an evaluation of the activities of these transcription factors is helpful in inferring their importance. We used a computational method called SCENIC to analyze the activity of the GRNs in each cell. *TFAP2D* has been detected in the developing mouse retina from E13.5 to E16.5 [68]. In the present study, *TFAP2D* and other transcription factors important for RGCs were detected in the developing human retina as early as 5 W. In human retina, *ONECUT1* and *ONECUT2* were active in developing HCs. In mice, *Onecut1* and *Onecut2* are expressed in both the neuroblast layer (NBL) and GCL at early stages (E11.5 and E12.5) [69]. Because of the high quality of our data set, the target genes of active transcription factors could be well defined. The functional annotations and dynamic expression patterns of active transcription factor target genes revealed major events during the development of retinal cells. Moreover, we verified transcription factors by immunofluorescence staining to explore the spatiotemporal gene expression network in the developing human retina. In conclusion, we identified various retinal cells in the human fetal NR. By exploring the expression patterns of transcription factors at both the transcriptome and protein levels, we provided important information about the spatiotemporal gene expression network in the developing human retina.

We labeled RGC axons with SNCG (which might be involved in modulating axonal architecture during development) at different stages to trace the states of RGCs during retinal development. RGCs were concentrated in the inner layer of the NR, and the density decreased during NR development. RGCs were globally generated in the direction from the optic nerves head toward the periphery and almost spread throughout the inner layer at approximately 9 W. However, in the present study, the sample size was too small to do a serious cell type analysis.

In the present study, RPE cells underwent sequential development with the decreased expression of *PAX6* and increased expression of visual cycle genes, such as *RPE65* and *LRAT*. We detected proliferating RPE cells at 5 and 6 W. In the present study, rhodopsin expression was first detected at 24 W, whereas genes related to visual cycle were already expressed in RPE cells as early as 13 W. In addition, the expression levels of genes related to photoreception, such as *RHO*, *SAG*, *GNGT1*, and *CNGA1*, were increased as PC differentiation proceeded. Moreover, using CellPhoneDB, we identified receptor–ligand pairs of RPE cells and PCs, such as *PTPRZ1* and *PTN*/*MDK*, *NGFR* and *TTR*, and *FLT1*/*NRP1*/*NRP2* and *VEGFA*, which also provided clues for RPE and PC interactions.

The ability to sense light requires interactions between PCs and RPE cells. The present study revealed the development of the NR and RPE, which will improve our understanding of neural development and provide clues about neural regeneration. The RPE is a single-layered epithelium and participates in the visual cycle [70], outer-segment phagocytosis process, and retinal-blood barrier [71]. The RPE is also associated with retinal degenerative diseases [72]. Thus, studies of the development of both the human NR and RPE are important. We dissected the transcriptome landscape of the developing human NR and RPE, as well as the inherited retinal disease map on human fetal retina. This study provides key information about human fetal retinal development, which is likely to be important for understanding and curing pathological conditions.

## Materials and methods

### Ethics statement

The present study was approved by the Reproductive Study Ethics Committee of Peking University Third Hospital (Research License 2012SZ-013 and 2017SZ-043). All donors signed the written informed consent and voluntarily donated aborted fetuses for the present study.

### Isolation of single cells from human NR or RPE

We collected fetal eyes from voluntarily donated aborted fetuses at 5 to 24 W of gestation. The NR and RPE were separately dissected under microscopes. Then, the NR or RPE tissues were digested into single cells using 2 mg/mL Collagenase/Dispase (Roche) by incubating them at 37 °C for approximately 5 min.

### Preparation of the single-cell RNA-seq library

The modified STRT protocol for multiplexed single-cell RNA-seq was described in previous studies [45–48]. Briefly, after digestion, single retinal cells were randomly selected and transferred into 2 μL of cell lysis buffer using a mouth pipette. The cell lysis buffer contained 0.1 U/μL RNase Inhibitor (TaKaRa, 2313B), 0.0475% Triton X-100 (Sigma-Aldrich, X100), 2.5 μM dNTP mixture (Thermo, R0193), and 2.5 μM barcode-reverse transcriptase (RT) primer (TCAGACGTGTGCTCTTCCGATCT-XXXXXXXX-NNNNNNNN-T25, with X representing the nucleotide of cell-specific barcodes and N representing the UMIs) (S9 Table). We thoroughly vortexed the tubes containing a single cell and the lysis buffer and incubated the tubes at 72 °C for 3 min to release the linearized RNA molecules and immediately placed the tubes on ice. The RT reaction was performed by adding 2.85 μL of RT mixture (40 U SuperScript II RT (Invitrogen, 18064071), 5 U of RNase Inhibitor, 5X Superscript II first-strand buffer, 25 mM DTT, 5 M betaine (Sigma-Aldrich, B0300), 30 mM MgCl_2_ (Sigma-Aldrich, 63020), and 1.75 μM TSO primer (AAGCAGTGGTATCAACGCAGAGTACATrGrG+G, rG represents riboguanosines one [+G] and +G indicates the LNA-modified guanosine) to each tube, and then we placed the tubes in a thermo cycler and incubated them at 25 °C for 5 min, 42 °C for 60 min, 50 °C for 30 min, and 70 °C for 10 min.

After the RT reaction, we added 7.5 μL of the PCR mixture (6.25 μL of 2× KAPA HiFi HotStart Ready Mix [KK2602] and 300 nM of ISPCR oligo [AAGCAGTGGTATCAACGCAGAGT], and 1 μM of 3’Anchor oligo [GTGACTGGAGTTCAGACGTGTGCTCTTCCGATC]) to each tube. The amplification was performed using the following program: 4 cycles at 98 °C for 20 s, 65 °C for 30 s, and 72 °C for 5 min, followed by 15 cycles at 98 °C for 20 s, 67 °C for 15 s, and 72 °C for 5 min, with a final cycle at 72 °C for 5 min.

Subsequently, we pooled the amplified samples with different cell barcodes together and purified these samples with DNA Clean & Concentrator-5 (Vistech, DC2005) and twice with 0.8X Ampure XP beads (Beckman, A63882).

Purified cDNAs were amplified with biotinylated index primer (Biotin/CAAGCAGAAGACGGCATACGAGATindexGTGACTGGAGTTCAGACGTGTGCTCTTCCGATC) and ISPCR oligo for 4–5 cycles. Biotinylated cDNAs were first purified and subsequently sheared into fragments of approximately 300 bp using Covaris. Next, we enriched the fragmented cDNAs, which were primed by biotinylation and contained barcodes and UMI sequences, using Dynabeads MyOne Streptavidin C1 (Invitrogen, 65002).

We constructed a library based on the enriched cDNA fragments, which were attached to the C1 beads, using KAPA Hyper Prep Kits (KK8505). We used the NEB U-shape adaptor for ligation. The final amplification was performed with the Illumina QP2 primer (CAAGCAGAAGACGGCATACGA) and the short universal primer (AATGATACGGCGACCACCGAGATCTACACTCTTTCCCTACACGAC) for seven cycles. Libraries were sequenced to generate 150-bp paired-end reads on an Illumina Hiseq4000 platform (sequenced by Novogene).

### Read alignment and calculation of the gene count

A custom script was used to trim the primer, TSO, and polyA sequences for the raw input reads. Additionally, the cell barcode was also extracted and input into the read.id information of the fastq file. Subsequently, the extracted reads were mapped to the hg38 genome using Hisat2 v2.0.5. The unique mapped reads were then counted by FeatureCounts v1.5.2 using the Ensembl release 84 GTF transcriptome annotation. Finally, we obtained the following table (Table 1):

**Table 1:** Transcriptome annotation table of each mapped read.

Batch	CellBacode	UMI	Gene
9 W_RET1	NCACAGAA	TTTTTTTT	ENSG00000111247
9 W_RET1	NAGGTACA	TTAAATAT	ENSG00000174744
9 W_RET1	NGTACAAG	GGATTTAT	ENSG00000124333
9 W_RET1	NCGCTCGA	TTTGTTAA	ENSG00000280234
9 W_RET1	NAACATCG	GTTTGTGA	ENSG00000100321

Abbreviation: UMI, unique molecular identifier.

Spark v1.6.2 was used to identify cells with at least 500 genes and then to cast the table into a sample (2,553)-gene (58,830) count matrix.

### Filtering of the low-quality cells and identification of the cell class using the dimension reduction method

We used the Seurat R/Bioconductor toolkit to perform the dimension reduction process. Gene expression levels were quantified as TPM, and the copy numbers of each transcript were quantified based on the number of distinct UMIs. Only cells expressing more than 1,000 genes and at least 10,000 transcripts were retained. Then, the variation for each gene was calculated, and only genes with a proper level of variation (fxn.x = expMean, fxn.y = logVarDivMean, x.low.cutoff = 0.5, x.high.cutoff = 10, y.cutoff = 0.5, and do.contour = F) were considered in subsequent analyses.

After two steps, 2,421 samples with 1,230 high-variable genes remained for the PCA. The top 30 principal components were used for the JackStraw analysis, and 18 principal components were ultimately used in the RunTSNE analysis. Finally, the cell group information was manually annotated for each cell based on the t-SNE clustering group information.

We used the Seurat function find_all_markers (test.use = "roc") to identify cluster-specific markers.

We used the R package Seurat to identify subclasses in one cluster.

### Find cell class–specific transcription factors

First, we overlapped the DEGs found by Seurat with transcription factors identified by SCENIC. Second, to ensure transcription factors active, we retained transcription factors that were active among more than 50% of cells in a particular cell class.

A detailed description of the SCENIC method is available at https://github.com/aertslab/SCENIC.

### Identification of the cell trajectory during the maturation of the human fetal retina

Monocle v2.3.2 was used to construct the trajectory landscape of RGC/RPE development. As for RGCs, we applied the extreme gradient boosting (xgboost) R package v0.7.0 to modify the cell trajectory. First, RGCs were labeled with the group number identified by t-SNE in the previous steps to select representative genes. Then, xgboost R package v0.7.0 was used to train a model that could identify these different t-SNE labels based on the expression level of all genes. After the training process, genes with the top 10 highest importance scores in this model were selected. Subsequently, the trajectory was built using the DDRTree algorithm of the Monocle package, and we obtained a pseudo–developmental time from the trajectory data. Finally, the expression levels of the selected genes were plotted using the genSmoothCurves function.

### Evaluate the detailed gene expression patterns of transcription factor target genes and RPE tendency genes

We applied the R package (cummeRbund) to group target genes of cell class–specific transcription factors using variable genesCluster (k = 3) and group RPE high-variable genes using variable genesCluster (k = 9).

### Cell–cell interaction analysis

We used a software called CellPhoneDB—which has a database of ligands, receptors, and their interaction—to systematically predict cell–cell interaction molecules with default settings (https://github.com/Teichlab/cellphonedb) [61]. For the top receptor–ligand pairs, we applied R package ggalluvial to visualize the interaction modules.

### Immunofluorescence staining of frozen sections

The eyes were fixed with 1–10 mL of 4% paraformaldehyde overnight at 4 °C. Eyes were dehydrated with 1–5 ml of a 20% sucrose solution overnight at 4 °C and embedded in Tissue-Tek O.C.T. Compound (Sakura #4583).

For immunofluorescence staining, 12-μm sections were dried at 55 °C for 30 min and washed three times (10 min each) with 0.1% Triton in PBS (PBT). Sections were then permeabilized with 0.5% Triton in PBS for 30 min at room temperature and incubated with blocking buffer (0.1% Triton and 10% donkey serum in PBS) for 90 min at room temperature. Primary antibodies were diluted in blocking buffer. Subsequently, sections were incubated with diluted primary antibodies overnight at 4 °C. After three washes (20 min each), sections were incubated with diluted secondary antibodies (1:400) at room temperature for 2 h. Finally, sections were washed three times (20 min each) and incubated with Prolong Gold Antifade Reagent with DAPI (Invitrogen #1846939). Images were acquired using a confocal laser scanning microscope (Leica TCS SP8, Leica Microsystems, Wetzlar, Germany).

The primary antibodies used in the present study were rabbit anti-SNCG (Abcam #ab55424, 1:500 dilution), mouse anti-RPE65 (Abcam #ab78036, 1:100 dilution), mouse anti-NEUROD1 (Abcam, #ab60704, 1:100 dilution), mouse anti-SOX2 (Abcam #ab79351, 1:100 dilution), rabbit anti-SOX2 (Abcam #ab59776, 1:100 dilution), rabbit anti-KI67 (Abcam #ab15580, 1:100 dilution), rabbit anti-BRN3B/POU4F2 (Abcam #ab56026, 1:100 dilution), and rabbit anti-AP2 beta (Abcam #ab221094, 1:100 dilution).

### GO analysis

We used the DAVID Functional Annotation tool [73] (version 6.8, https://david.ncifcrf.gov/) to do gene-enrichment analysis. The *P* value listed after GO term in this paper is the EASE Score, a modified Fisher exact *P* value.

### RNAscope in situ hybridization

We applied RNAscope assay [74] to validate RNA detected in our single-cell RNA-seq data. Briefly, fresh human fetal retinas were fixed for at least 24 h in 4% paraformaldehyde (with DEPC) and proceeded to dehydrate with graded concentration of sucrose. After embedding with O.C.T. compound, 10-μm retina frozen sections were obtained for the following mRNA detection. According to the instructions provided by commercial RNAscope Multiplex Fluorescent Reagent Kit (Advanced Cell Diagnostics), the target probes *ONECUT2* and *NTRK1*, which were synthesized by Advanced Cell Diagnostics, were hybridized to the retina sections. After hybridization, mRNA signal was amplified and then labeled with TSA Plus Fluorescein. Images were acquired through Nikon A1RSi+ confocal microscope.

### Building the single-cell RNA-seq results browser

Codes for building the single-cell RNA-seq results browser (http://49.4.93.68:30004/) were deposed at https://github.com/huboqiang/singlecellbrowser. The needed files are S5 Table of this paper and GSE107618_Merge.TPM.csv.gz, which has been uploaded in GEO: GSE107618.

## Supporting information

S1 Fig Sampling information for each tissue and quality control of the data set. (A) Sampling information for the NR and RPE. (B) Plot of the distribution of the number of cells (y-axis) versus the number of genes detected. (C) Plot of the distribution of the number of cells (y-axis) versus the number of gene UMIs detected. (D) Embryo information mapped on t-SNE plot to evaluate the batch effect. NR, neural retina; RPE, retinal pigment epithelium; t-SNE, t-distributed stochastic neighbor embedding; UMI, unique molecular identifier; W, week.
(PDF)

S2 Fig Identification of retinal cells. (A) The clusters need to be reclustering. Clusters are indicated with different colors. (B) Subclasses of cells in cluster 3. (C) Representative genes that distinguish each RPC subclass. (D) Subclasses of cells in cluster 10. (E) Representative genes that distinguish each cluster 10 subclass. (F) Subclasses of cells in cluster 16. (G) Representative genes that distinguish BCs from PCs in cluster 16. (H) Subclasses of cells in cluster 3. (I) Representative genes that distinguish ACs from BCs in cluster 3. (J) Heatmap showing the differentially expressed genes of RPC subclasses and Müller glia cells. AC, amacrine cell; BC, bipolar cell; PC, photoreceptor cell; RPC, retinal progenitor cell; W, week.
(PDF)

S3 Fig Validation of markers by RNAscope assay. RNAScope detection of *ONECUT2* and *NTRK1* in 17-W and 23-W human retina. W, week.
(PDF)

S4 Fig Cell cycle state of cells detected in the present study. (A) Density map showing the cell cycle state of microglia, Müller glia cells, and fibroblast cells in each period. (B) Heatmaps showing the proliferative activity of each cell class. The *P* value of the one-tailed *t* test (greater) for the relative expression level associated with gene sets associated with G1/S phases between cells within a certain stage/class and all cells. (C) Heatmaps showing the proliferative activity of each cell class. The *P* value of the one-tailed *t* test (greater) for the relative expression level associated with gene sets associated with G2/M phases between cells within a certain stage/class and all cells.
(PDF)

S5 Fig The expression of representative transcription factors in the human retina. (A) Regulon matrix-based t-SNE plot showing the cell classes detected in our study. (B) The expression of *SOX2*, *POU4F2*, *TFAP2B*, and *NEUROD1* is mapped on the t-SNE plot. (C) Expression of the SOX2 and NEUROD1 proteins in the human fetal retina. White bars represent 50 μm in (C1-C3), 25 μm in (C4-C6), and 100 μm in (C7-C9). (D) Higher-magnification images of selected regions shown in (C). Representative cells are indicated by different colored arrows. (E) The expression of BRN3B (gene *POU4F2*) and NeuroD1 proteins in the human fetal retina. White bars represent 25 μm in E1-E3 and 50 μm in E4-E6. (F) Higher-magnification images of selected regions shown in (E). Representative cells are indicated by different colored arrows. (G) The expression of TFAP2B and SOX2 proteins in the human fetal retina. White bars represent 25 μm. t-SNE, t-distributed stochastic neighbor embedding; W, week.
(PDF)

S6 Fig Dynamic changes in the transcriptome of RGCs. (A) Smoothed expression level of cell trajectory marker genes in RGCs along the pseudotime calculated by Monocle2. (B) Pseudo–time plots of representative genes, whose expression is dynamically changed during RGC development. Sampling stages are shown in different colors. RGC, retinal ganglion cell; W, week.
(PDF)

S7 Fig RPE high-variable genes groups and states of PCs. (A) Smoothed expression levels of cell trajectory marker genes in RPE cells along the pseudotime calculated by Monocle2. (B) PCA plot of PCs using genes with a high variance in their expression levels, identified by Seurat. These results were further divided into three clusters using the KMeans algorithm. Groups are indicated by shape, and stages are indicated by color. (C) Expression levels (log[TPM]) of genes with high contributions to the principle components. PC, photoreceptor cell; PCA, principal component analysis; RPE, retinal pigment epithelium; TPM, transcripts per million; W, week.
(PDF)

S8 Fig Maps of other inherited retinal disease–related genes in human fetal retinal cells. Heatmap showing the expression patterns of other inherited retinal disease–related genes in human fetal retinal cells.
(PDF)

S9 Fig The expression patterns of human adult retinal cells in our data set. (A) The expression patterns of human adult retinal cells marker genes in our data set. (B) Comparison of fetal single-cell RNA-seq datasets from present and fetal bulk RNA-seq datasets from the previously published 2017 study by Hoshino and colleagues [38]. D, fetal day; RNA-seq, RNA sequencing; W, week.
(PDF)

S10 Fig The specificity of each human fetal retinal cell class–enriched gene in retinas of macaque and mouse. The average log fold change of each human fetal retinal cell class–enriched genes was showed across species. The average log fold change of these genes in macaque and mouse was determined by Peng and colleagues, 2019 [67], and Macosko and colleagues, 2015 [40]. The average log fold change of these genes in any macaque and mouse retinal cell subtypes was displayed.
(PDF)

S1 Table PCA gene and GO of two tissues. GO, gene ontology; PCA, principal component analysis.
(XLSX)

S2 Table Markers of each cluster. (XLSX)

S3 Table The results of GO analysis of each cluster. GO, gene ontology.
(XLSX)

S4 Table Markers of reclustered clusters and DEGs of all cell classes. DEG, differentially expressed gene.
(XLSX)

S5 Table The matrix of cell class on the t-SNE map. t-SNE, t-distributed stochastic neighbor embedding.
(XLSX)

S6 Table Cell cycle gene list. (XLSX)

S7 Table Genes and their importance scores found by model training using xgboost to identify any different RGCs. RGC, retinal ganglion cell.
(XLSX)

S8 Table RPE monocle tendency gene list. RPE, retinal pigment epithelium.
(XLSX)

S9 Table Barcode-RT primer information. RT, reverse transcriptase.
(XLSX)

S1 Data Numerical data for all figures. (XLSX)

## Abbreviations

AC: amacrine cell
AMD: age-related macular degeneration
BC: bipolar cell
bHLH: basic helix-loop-helix
CRALBP: cellular retinaldehyde binding protein
D: fetal day
DDRTree: discriminative dimensionality reduction tree
DEG: differentially expressed gene
E: embryonic day
GCL: ganglion cell layer
GO: gene ontology
GRN: gene regulatory network
HC: horizontal cell
INL: inner nuclear layer
IPM: interphotoreceptor matrix
KEGG: Kyoto Encyclopedia of Genes and Genomes
MDK: Midkine
NBL: neuroblast layer
NR: neural retina
ONL: outer nuclear layer
PC: photoreceptor cell
PCA: principal component analysis
PTN: pleiotrophin
*PTPRZ1*: receptor protein tyrosine phosphatase type Z
RGC: retinal ganglion cell
RNA-seq: RNA sequencing
RPC: retinal progenitor cell
RPE: retinal pigment epithelium
SCENIC: single-cell regulatory network inference and clustering
SNCG: gamma synuclein
STRT: single-cell tagged reverse transcription
TPM: transcripts per million
t-SNE: t-distributed stochastic neighbor embedding
UMI: unique molecular identifier
W: week
