# Supplementary material for: Dissecting the transcriptome landscape of the human fetal neural retina and retinal pigment epithelium by single-cell RNA-seq analysis
Source: PLoS Biol. 2019 Jul 3;17(7):e3000365. doi: 10.1371/journal.pbio.3000365 (PMC6634428; doi:10.1371/journal.pbio.3000365)

A

Stage	5W	6W		7W			8W			9W	
Embryo	embryo#1	embryo#1	embryo#2	embryo#1	embryo#2	embryo#3	embryo#1	embryo#2	embryo#3	embryo#1	embryo#2
Retinal Pigment Epithelium	48	48	48	48	24	0	48	43	96	25	48
Neural Retina	48	96	48	80	64	48	96	53	192	72	96
Total	96	144	96	128	88	48	144	96	288	97	144

Stage	11W	13W		17W		23W		24W	Total
Embryo	embryo#1	twin#1	twin#2	embryo#1	embryo#2	embryo#1	embryo#2	embryo#1	19
Retinal Pigment Epithelium	48	48	48	40	40	40	64	40	844
Neural Retina	96	96	88	80	96	96	96	96	1,637
Total	144	144	136	120	136	136	160	136	2,481

B

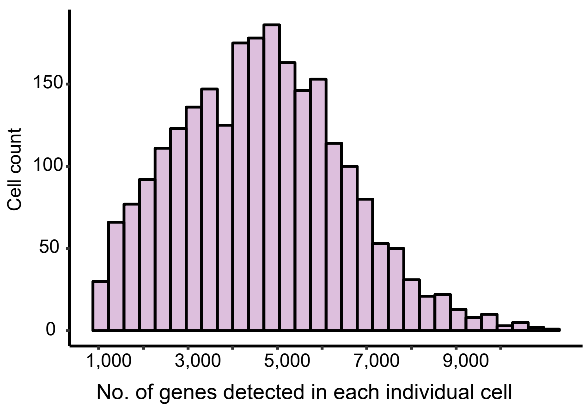

C

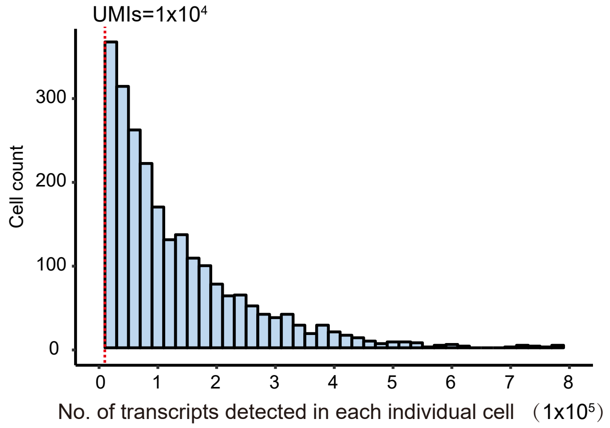

D

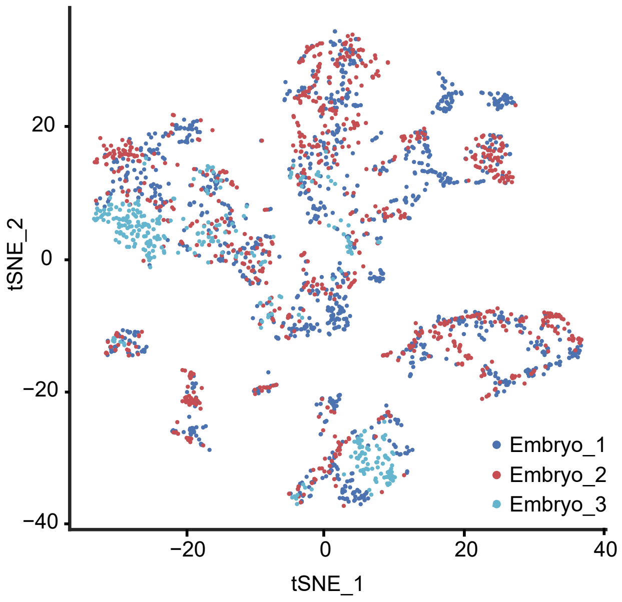

Supplement: S1 Fig — (A) Sampling information for the NR and RPE. (B) Plot of the distribution of the number of cells (y-axis) versus the number of genes detected. (C) Plot of the distribution of the number of cells (y-axis) versus the number of gene UMIs detected. (D) Embryo information mapped on t-SNE plot to evaluate the batch effect. NR, neural retina; RPE, retinal pigment epithelium; t-SNE, t-distributed stochastic neighbor embedding; UMI, unique molecular identifier; W, week. (PDF) [file pbio.3000365.s001.pdf]
