# Supplementary figures and images for: Dissecting the transcriptome landscape of the human fetal neural retina and retinal pigment epithelium by single-cell RNA-seq analysis
Source: PLoS Biol. 2019 Jul 3;17(7):e3000365. doi: 10.1371/journal.pbio.3000365 (PMC6634428; doi:10.1371/journal.pbio.3000365)

**A**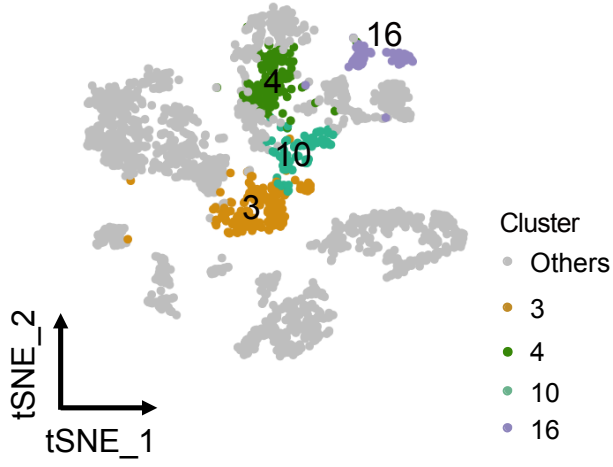**J**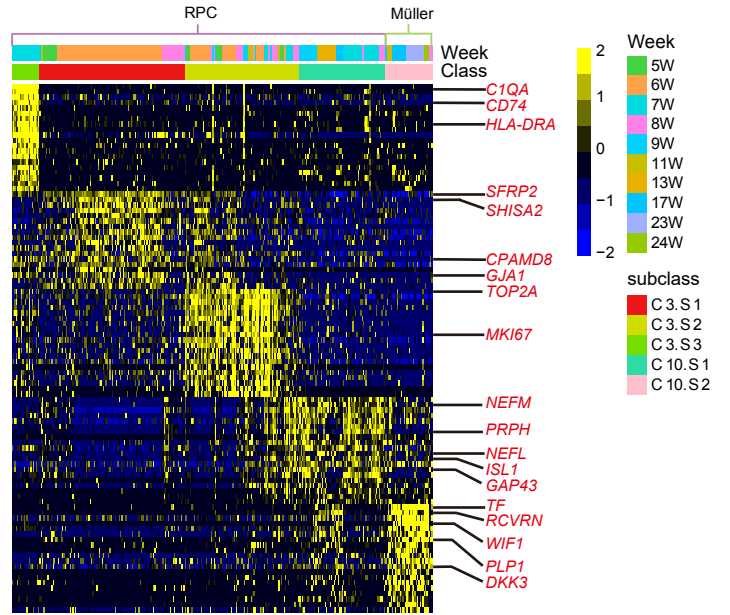**B**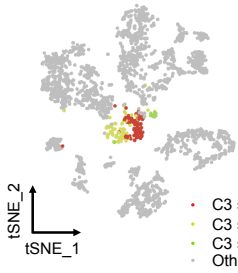**D**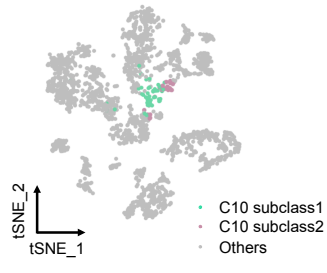**F**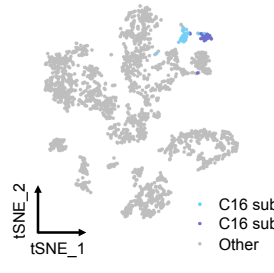**H**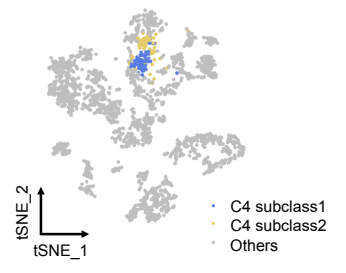**C**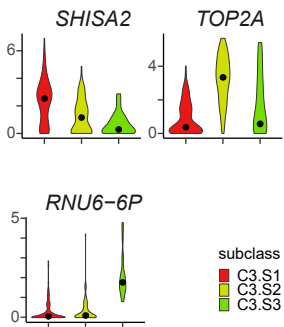**E**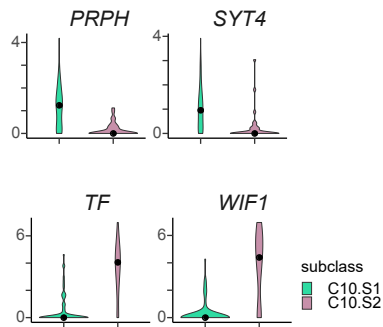**G**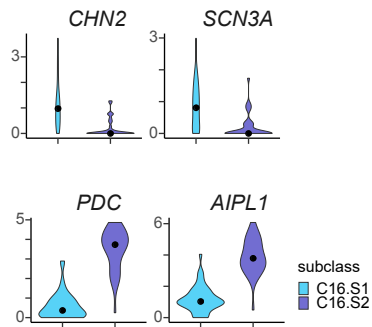**I**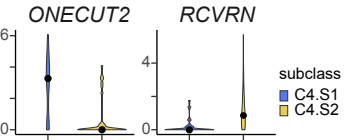

Supplement: S2 Fig — (A) The clusters need to be reclustering. Clusters are indicated with different colors. (B) Subclasses of cells in cluster 3. (C) Representative genes that distinguish each RPC subclass. (D) Subclasses of cells in cluster 10. (E) Representative genes that distinguish each cluster 10 subclass. (F) Subclasses of cells in cluster 16. (G) Representative genes that distinguish BCs from PCs in cluster 16. (H) Subclasses of cells in cluster 3. (I) Representative genes that distinguish ACs from BCs in cluster 3. (J) Heatmap showing the differentially expressed genes of RPC subclasses and Müller glia cells. AC, amacrine cell; BC, bipolar cell; PC, photoreceptor cell; RPC, retinal progenitor cell; W, week. (PDF) [file pbio.3000365.s002.pdf]

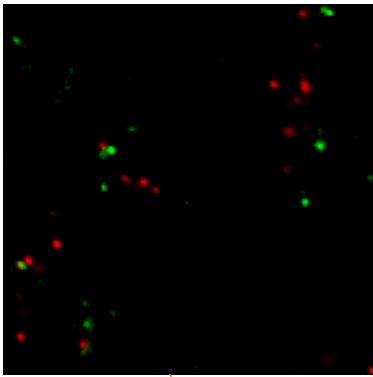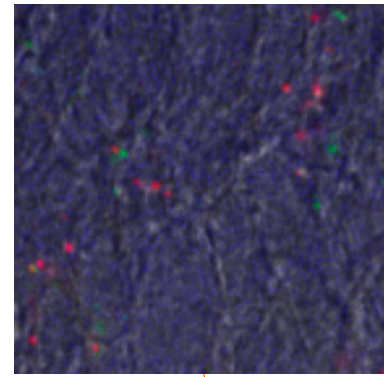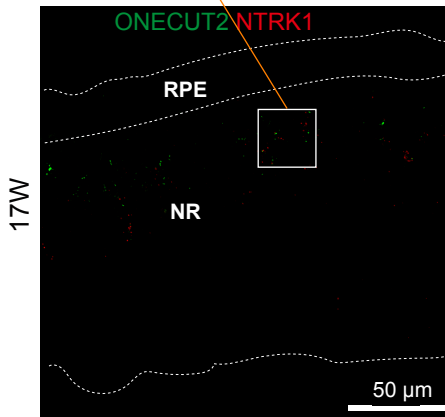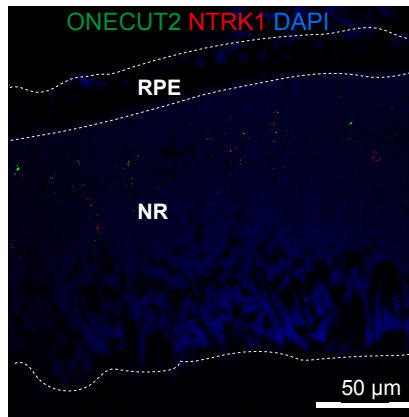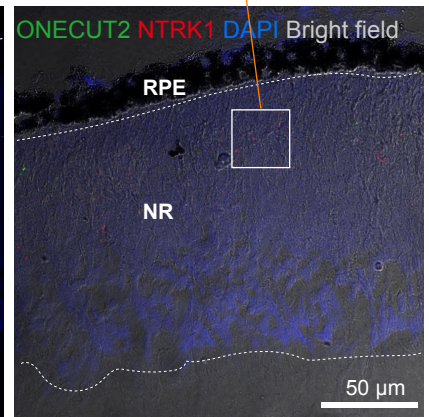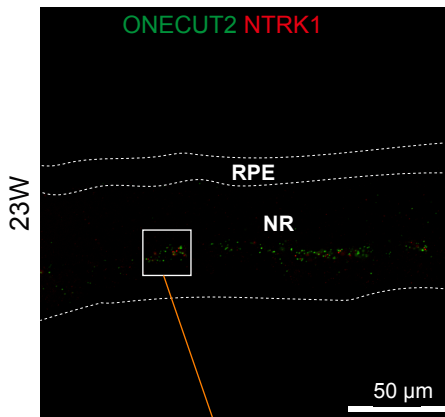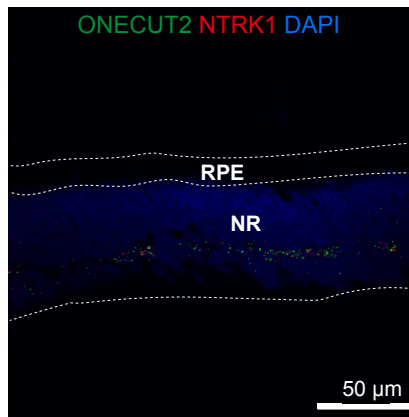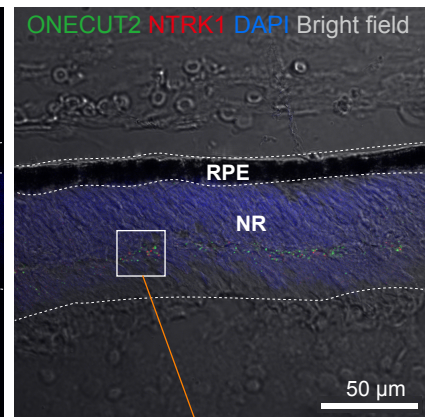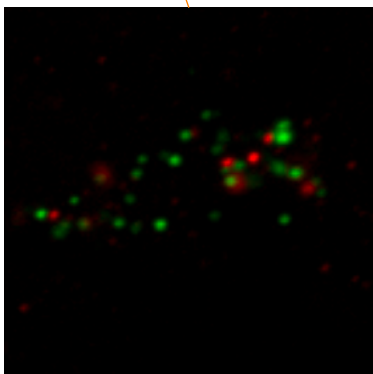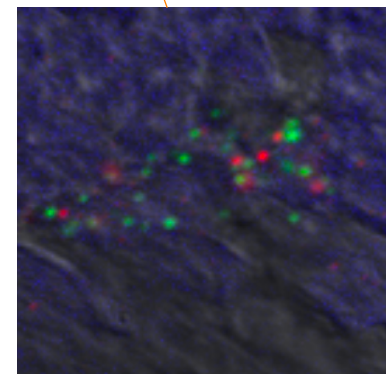

Supplement: S3 Fig — RNAScope detection of ONECUT2 and NTRK1 in 17-W and 23-W human retina. W, week. (PDF) [file pbio.3000365.s003.pdf]

**A**

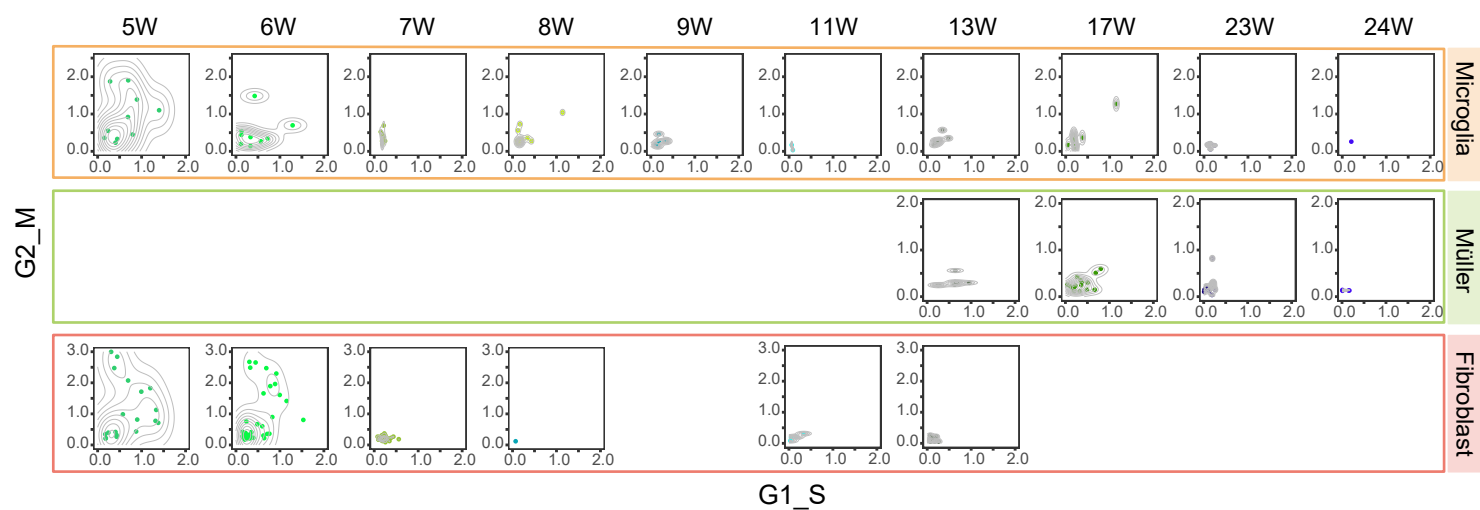

**B**

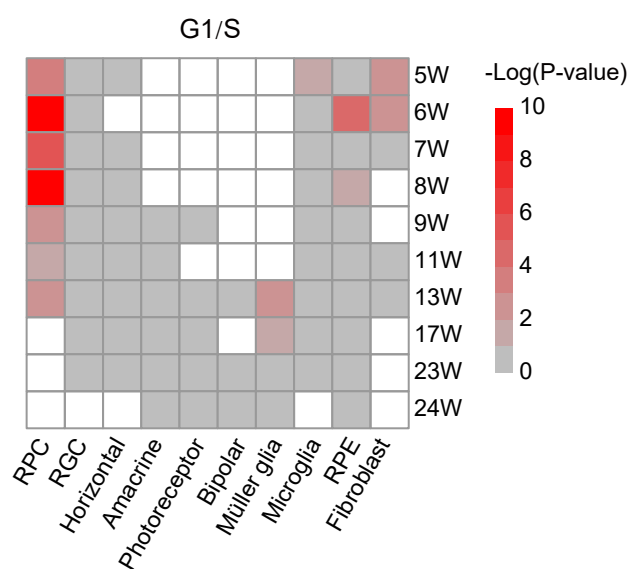

**C**

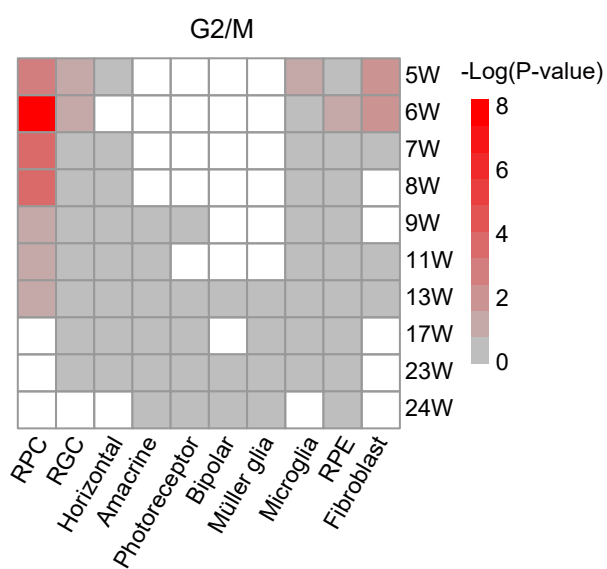

Supplement: S4 Fig — (A) Density map showing the cell cycle state of microglia, Müller glia cells, and fibroblast cells in each period. (B) Heatmaps showing the proliferative activity of each cell class. The P value of the one-tailed t test (greater) for the relative expression level associated with gene sets associated with G1/S phases between cells within a certain stage/class and all cells. (C) Heatmaps showing the proliferative activity of each cell class. The P value of the one-tailed t test (greater) for the relative expression level associated with gene sets associated with G2/M phases between cells within a certain stage/class and all cells. (PDF) [file pbio.3000365.s004.pdf]

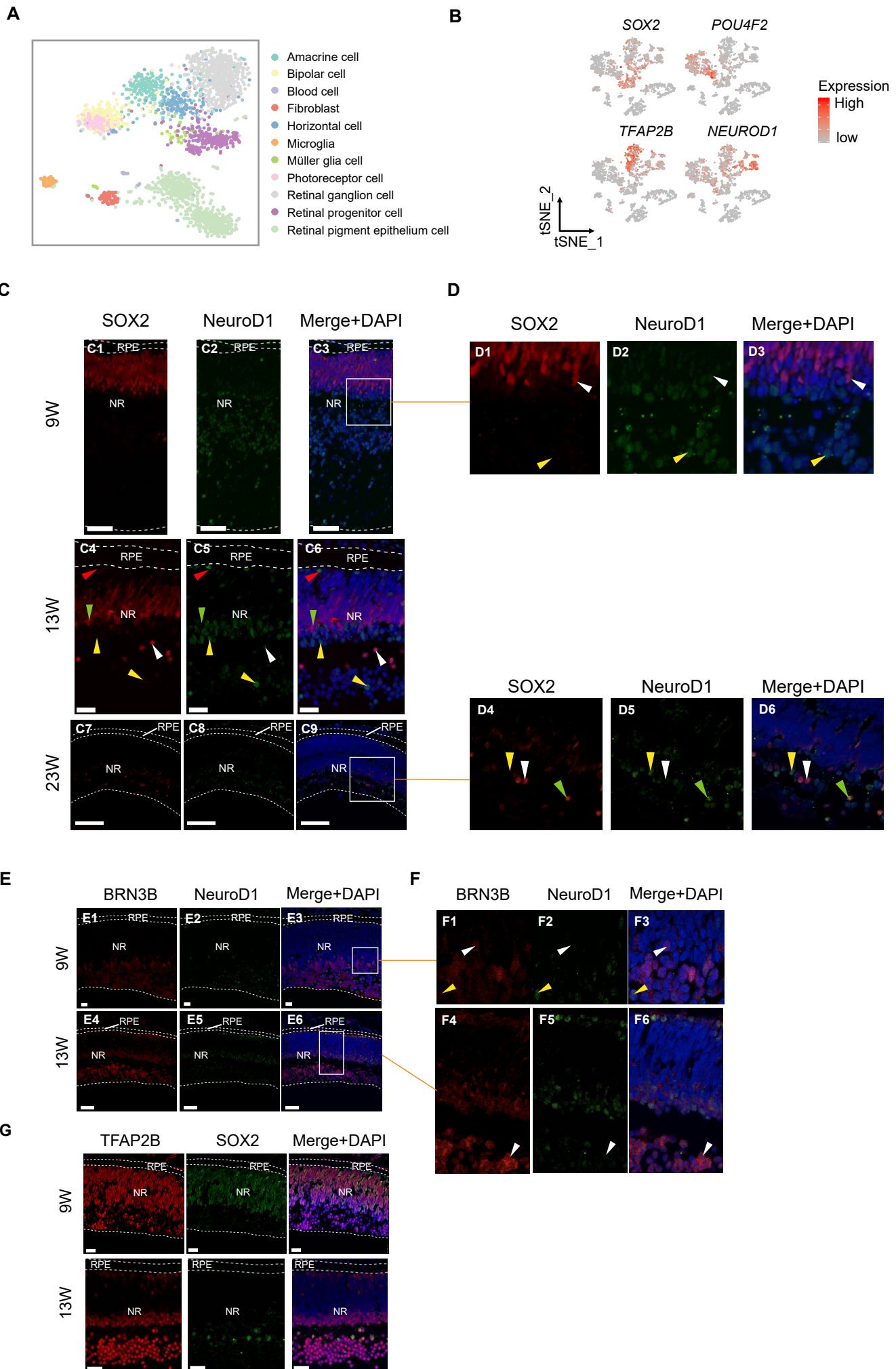

Supplement: S5 Fig — (A) Regulon matrix-based t-SNE plot showing the cell classes detected in our study. (B) The expression of SOX2, POU4F2, TFAP2B, and NEUROD1 is mapped on the t-SNE plot. (C) Expression of the SOX2 and NEUROD1 proteins in the human fetal retina. White bars represent 50 μm in (C1-C3), 25 μm in (C4-C6), and 100 μm in (C7-C9). (D) Higher-magnification images of selected regions shown in (C). Representative cells are indicated by different colored arrows. (E) The expression of BRN3B (gene POU4F2) and NeuroD1 proteins in the human fetal retina. White bars represent 25 μm in E1-E3 and 50 μm in E4-E6. (F) Higher-magnification images of selected regions shown in (E). Representative cells are indicated by different colored arrows. (G) The expression of TFAP2B and SOX2 proteins in the human fetal retina. White bars represent 25 μm. t-SNE, t-distributed stochastic neighbor embedding; W, week. (PDF) [file pbio.3000365.s005.pdf]

**A**

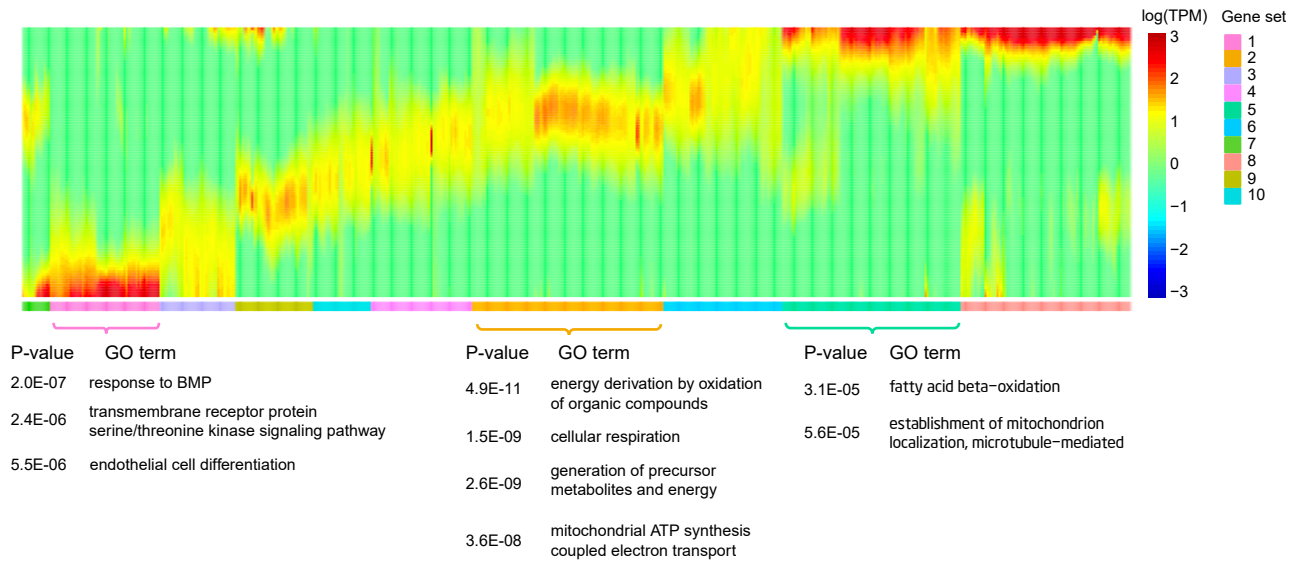

**B**

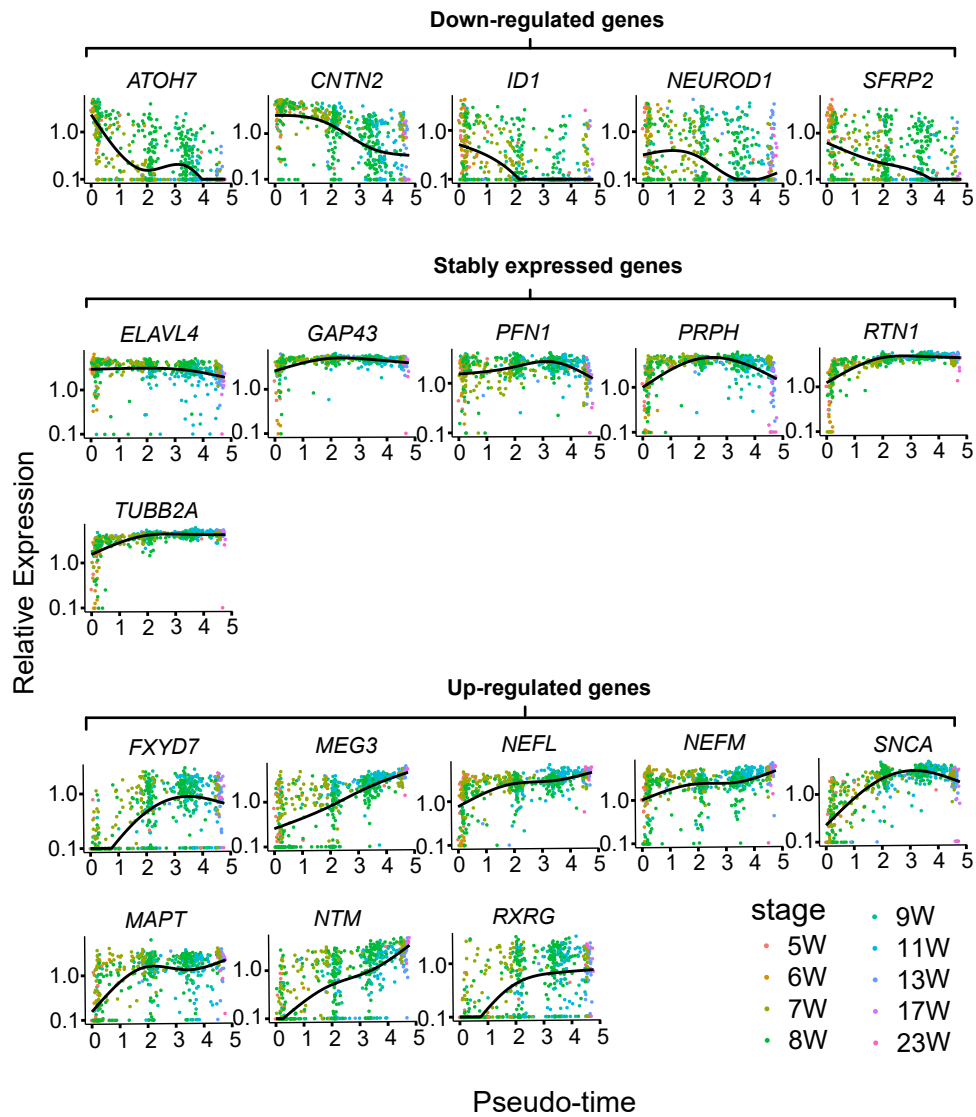

Supplement: S6 Fig — (A) Smoothed expression level of cell trajectory marker genes in RGCs along the pseudotime calculated by Monocle2. (B) Pseudo–time plots of representative genes, whose expression is dynamically changed during RGC development. Sampling stages are shown in different colors. RGC, retinal ganglion cell; W, week. (PDF) [file pbio.3000365.s006.pdf]

**A**

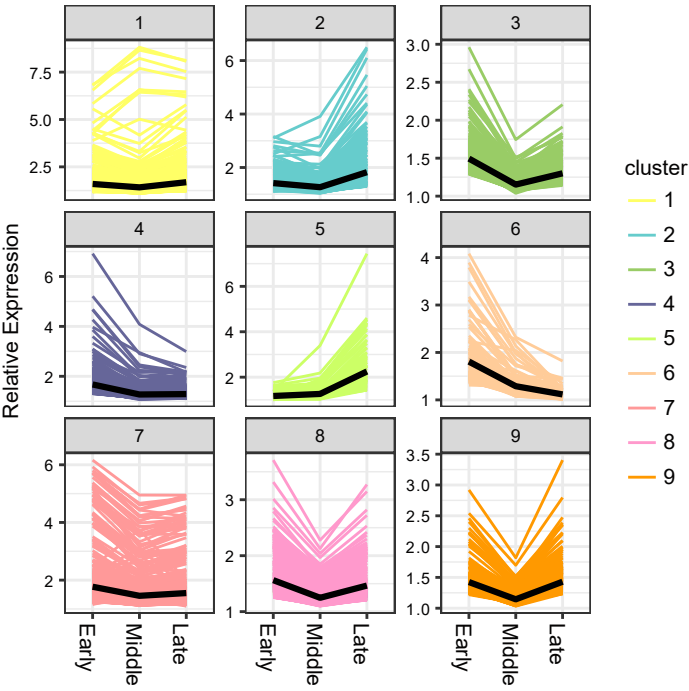

**B**

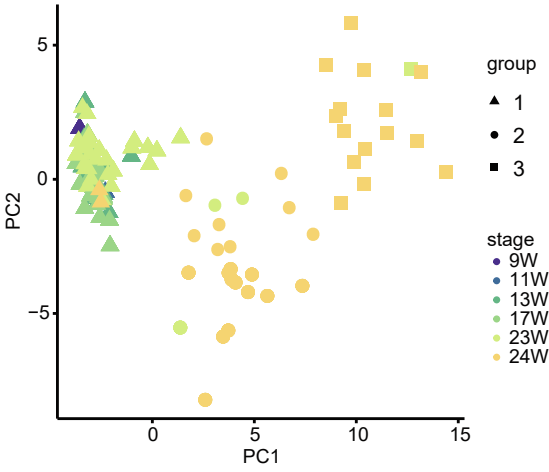

**C**

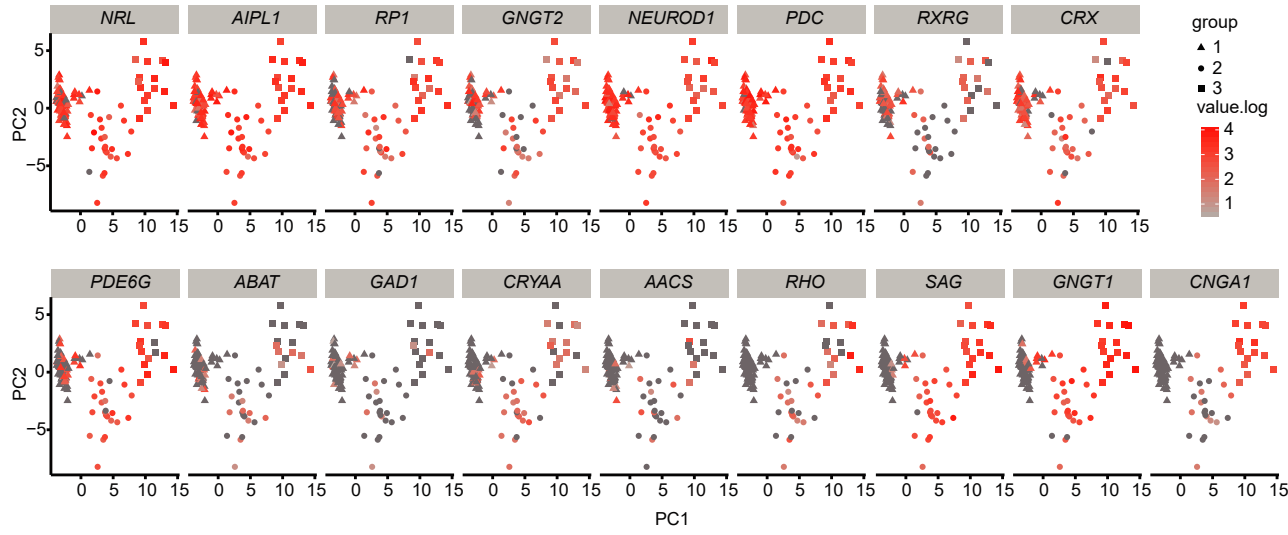

Supplement: S7 Fig — (A) Smoothed expression levels of cell trajectory marker genes in RPE cells along the pseudotime calculated by Monocle2. (B) PCA plot of PCs using genes with a high variance in their expression levels, identified by Seurat. These results were further divided into three clusters using the KMeans algorithm. Groups are indicated by shape, and stages are indicated by color. (C) Expression levels (log[TPM]) of genes with high contributions to the principle components. PC, photoreceptor cell; PCA, principal component analysis; RPE, retinal pigment epithelium; TPM, transcripts per million; W, week. (PDF) [file pbio.3000365.s007.pdf]
