# Supplementary material for: Dissecting the transcriptome landscape of the human fetal neural retina and retinal pigment epithelium by single-cell RNA-seq analysis
Source: PLoS Biol. 2019 Jul 3;17(7):e3000365. doi: 10.1371/journal.pbio.3000365 (PMC6634428; doi:10.1371/journal.pbio.3000365)
Supplement: S8 Fig — Heatmap showing the expression patterns of other inherited retinal disease–related genes in human fetal retinal cells. (PDF) [file pbio.3000365.s008.pdf]

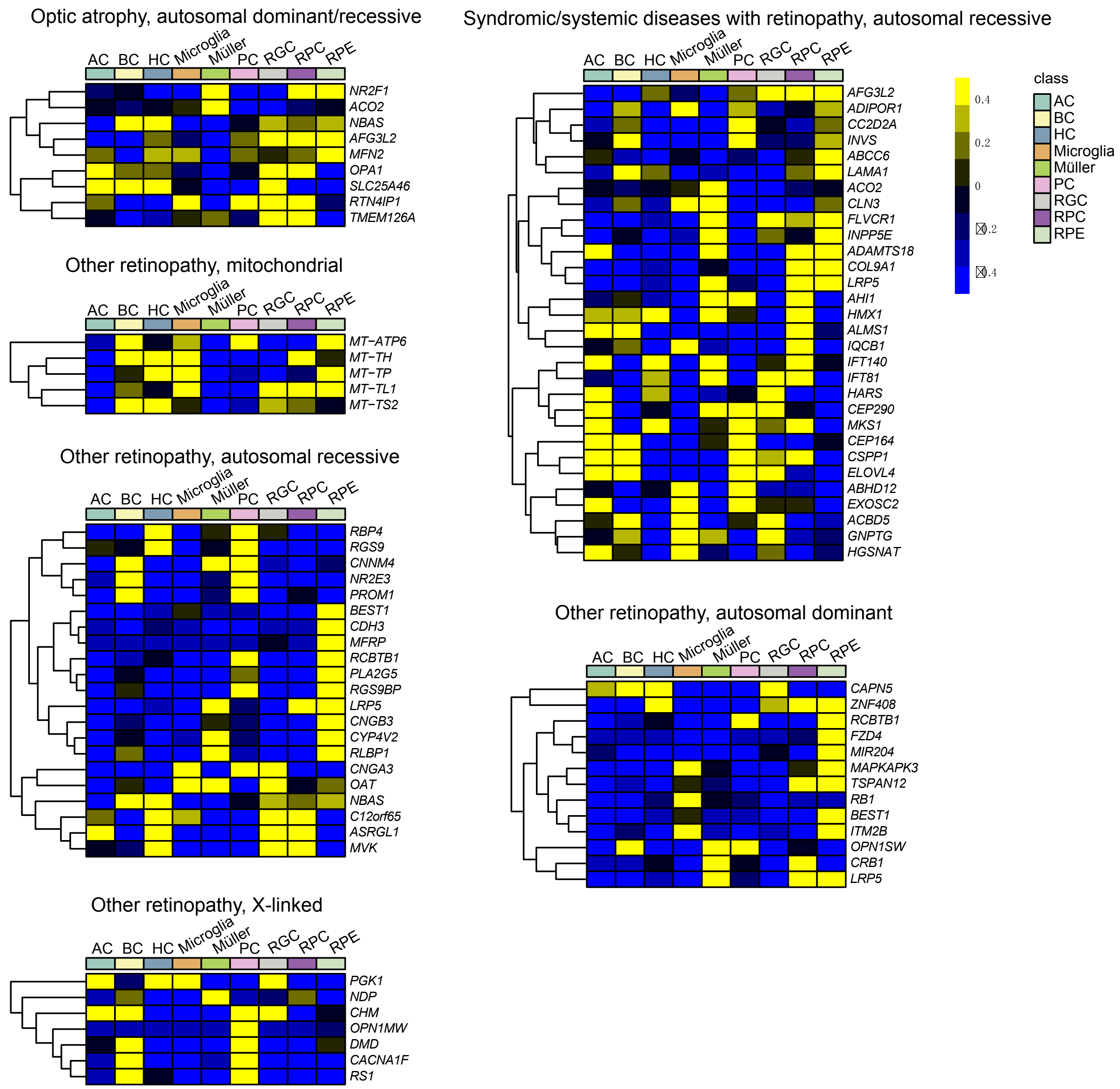
Optic atrophy, autosomal dominant/recessive
AC BC HC Microglia Müller PC RGC RPC RPE
NR2F1
ACO2
NBAS
AFG3L2
MFN2
OPA1
SLC25A46
RTN4IP1
TMEM126A
Other retinopathy, mitochondrial
AC BC HC Microglia Müller PC RGC RPC RPE
MT−ATP6
MT−TH
MT−TP
MT−TL1
MT−TS2
Other retinopathy, autosomal recessive
AC BC HC Microglia Müller PC RGC RPC RPE
RBP4
RGS9
CNNM4
NR2E3
PROM1
BEST1
CDH3
MFRP
RCBTB1
PLA2G5
RGS9BP
LRP5
CNGB3
CYP4V2
RLBP1
CNGA3
OAT
NBAS
C12orf65
ASRGL1
MVK
Other retinopathy, X-linked
AC BC HC Microglia Müller PC RGC RPC RPE
PGK1
NDP
CHM
OPN1MW
DMD
CACNA1F
RS1
Syndromic/systemic diseases with retinopathy, autosomal recessive
AC BC HC Microglia Müller PC RGC RPC RPE
AFG3L2
ADIPOR1
CC2D2A
INVS
ABCC6
LAMA1
ACO2
CLN3
FLVCR1
INPP5E
ADAMTS18
COL9A1
LRP5
AHI1
HMX1
ALMS1
IQCB1
IFT140
IFT81
HARS
CEP290
MKS1
CEP164
CSPP1
ELOVL4
ABHD12
EXOSC2
ACBD5
GNPTG
HGSNAT
0.4
0.2
0
class
AC
BC
HC
Microglia
Müller
PC
RGC
RPC
RPE
Other retinopathy, autosomal dominant
AC BC HC Microglia Müller PC RGC RPC RPE
CAPN5
ZNF408
RCBTB1
FZD4
MIR204
MAPKAPK3
TSPAN12
RB1
BEST1
ITM2B
OPN1SW
CRB1
LRP5
