# Supplementary material for: Dissecting the transcriptome landscape of the human fetal neural retina and retinal pigment epithelium by single-cell RNA-seq analysis
Source: PLoS Biol. 2019 Jul 3;17(7):e3000365. doi: 10.1371/journal.pbio.3000365 (PMC6634428; doi:10.1371/journal.pbio.3000365)
Supplement: S9 Fig — (A) The expression patterns of human adult retinal cells marker genes in our data set. (B) Comparison of fetal single-cell RNA-seq datasets from present and fetal bulk RNA-seq datasets from the previously published 2017 study by Hoshino and colleagues [38]. D, fetal day; RNA-seq, RNA sequencing; W, week. (PDF) [file pbio.3000365.s009.pdf]

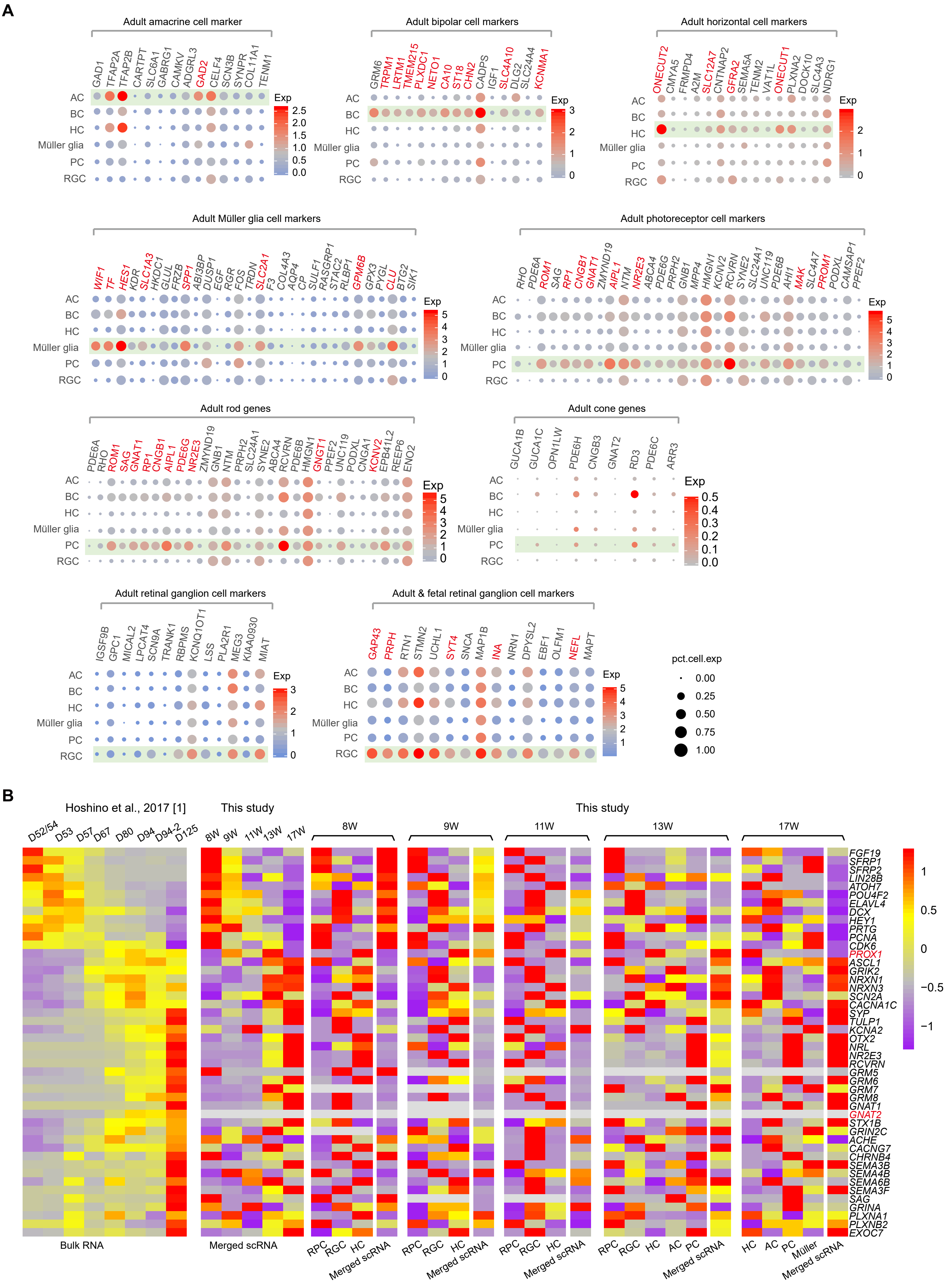

Reference

1. Hoshino A, Ratnapriya R, Brooks MJ, Chaitankar V, Wilken MS, Zhang C, et al. Molecular Anatomy of the Developing Human Retina. Developmental cell. 2017;43(6):763-79. e4.
