# Supplementary material for: Dissecting the transcriptome landscape of the human fetal neural retina and retinal pigment epithelium by single-cell RNA-seq analysis
Source: PLoS Biol. 2019 Jul 3;17(7):e3000365. doi: 10.1371/journal.pbio.3000365 (PMC6634428; doi:10.1371/journal.pbio.3000365)
Supplement: S10 Fig — The average log fold change of each human fetal retinal cell class–enriched genes was showed across species. The average log fold change of these genes in macaque and mouse was determined by Peng and colleagues, 2019 [67], and Macosko and colleagues, 2015 [40]. The average log fold change of these genes in any macaque and mouse retinal cell subtypes was displayed. (PDF) [file pbio.3000365.s010.pdf]

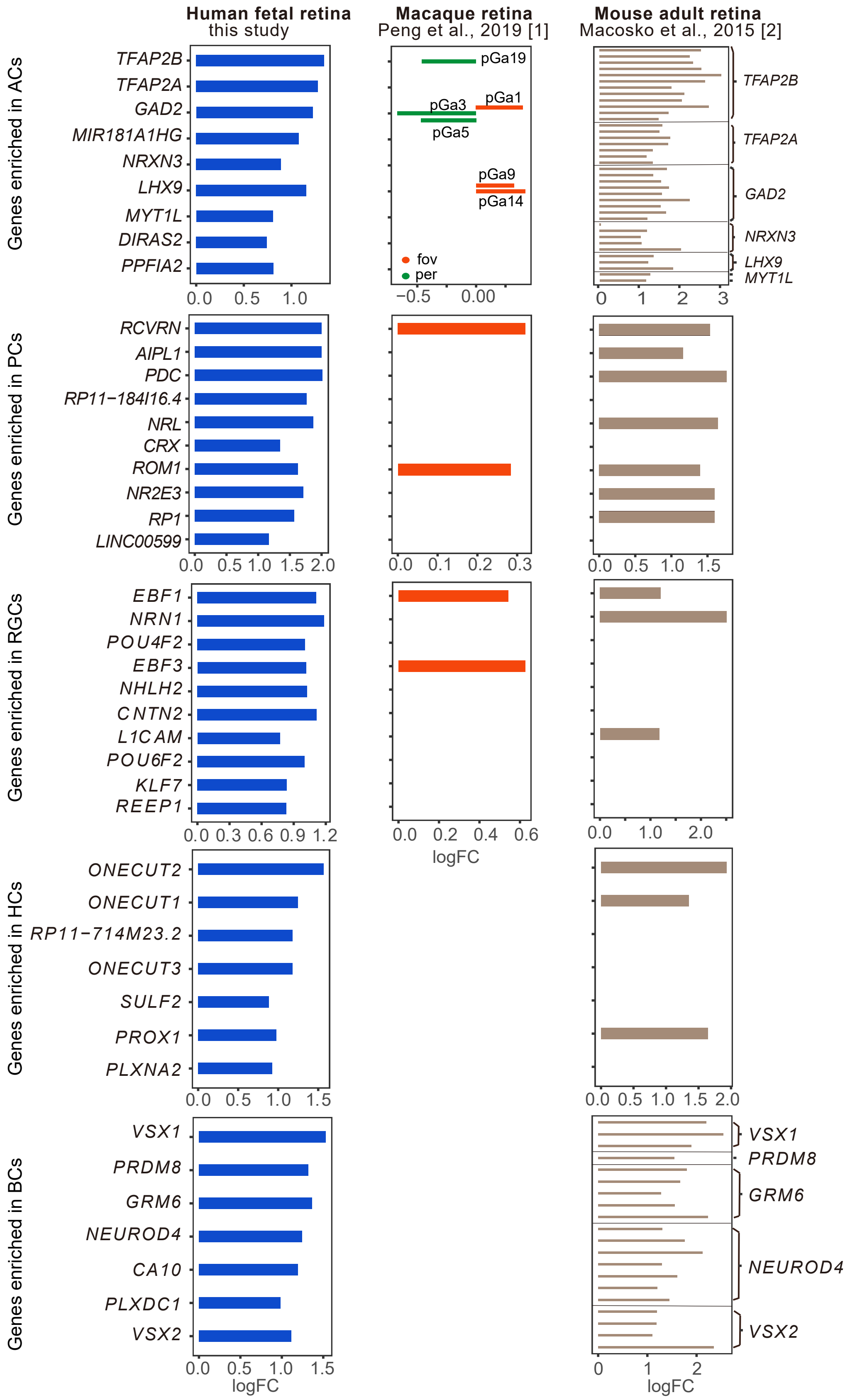

Reference

1. Peng Y-R, Shekhar K, Yan W, Herrmann D, Sappington A, Bryman GS, et al. Molecular classification and comparative taxonomics of foveal and peripheral cells in primate retina. Cell. 2019;176(5):1222-37. e22.

2. Macosko EZ, Basu A, Satija R, Nemesh J, Shekhar K, Goldman M, et al. Highly parallel genome-wide expression profiling of individual cells using nanoliter droplets. Cell. 2015;161(5):1202-14.
